# Designing Moderately‐Solvating Electrolytes for High‐Performance Lithium–Sulfur Batteries

**DOI:** 10.1002/adma.202503365

**Published:** 2025-06-04

**Authors:** David J. Kautz, Xia Cao, Peiyuan Gao, Shuo Feng, Qian Zhao, Saurabh Parab, Yaobin Xu, Joseph P. Quinn, Muhammad Mominur Rahman, Sha Tan, Xin Zhang, Sanaz Ketabi, Aqsa Nazir, Junxia Wang, Fang Dai, Shen Wang, Dongping Lu, Enyuan Hu, Y. Shirley Meng, Chongmin Wang, Jun Liu, Ji‐Guang Zhang, Wu Xu

**Affiliations:** ^1^ Energy and Environment Directorate Pacific Northwest National Laboratory Richland WA 99354 USA; ^2^ Physical and Computational Sciences Directorate Pacific Northwest National Laboratory Richland WA 99354 USA; ^3^ Environmental Molecular Sciences Laboratory Pacific Northwest National Laboratory Richland WA 99354 USA; ^4^ Department of Nano Engineering University of California San Diego La Jolla CA 92093 USA; ^5^ Chemistry Division Brookhaven National Laboratory Upton NY 11973 USA; ^6^ General Motors Research and Development Center Warren MI 48092 USA; ^7^ Materials Science and Engineering Department University of Washington Seattle WA 98195 USA

**Keywords:** cycle and calendar life, lithium‐sulfur battery, moderately solvating electrolyte, polysulfide, self‐discharge

## Abstract

New electrolytes are critical for high‐energy lithium (Li)–sulfur (S) batteries (LSBs) to ensure their stability against Li metal anode and polysulfides (PSs) shuttling which hinder the large‐scale application of LSBs. In this study, the design principle of moderately solvating electrolytes (MSEs) for LSBs is demonstrated by using a multiple‐solvent system comprising of a highly solvating solvent, a weakly solvating solvent, and a non‐solvating solvent to create a well‐balanced electrolyte system. This resulting electrolyte significantly improves the cycle life of LSBs, achieving 300 cycles, which is twice as long as that of similar cells with the conventional electrolyte and it also ensures stable calendar life for at least seven months. The optimal MSE forms robust passivation layers enhancing the structural integrity of both S and Li metal electrodes after cycling. These virtues effectively hinder parasitic side reactions and self‐discharge behavior of LSBs. This electrolyte design principle is versatile and can be applied to other battery chemistries, providing a potential path toward the development of a more efficient and stable battery system. By addressing key challenges such as the instability of electrodes and shuttling of polysulfides, this electrolyte approach offers promising solutions for advancing LSB technology.

## Graphical abstract

The design principle of moderately solvating electrolytes (MSEs) for lithium–sulfur batteries (LSBs) is demonstrated by using a multiple‐solvent system comprising of a highly‐solvating solvent, a weakly‐solvating solvent, and a non‐solvating solvent. The optimal MSE significantly improves the cycle life of LSBs by doubling that of similar cells with the conventional electrolyte and enables stable calendar life for at least seven months.

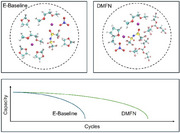

## 1 Introduction

Lithium (Li)–sulfur (S) batteries (LSBs) are widely regarded as a promising candidate for next‐generation batteries due to their high theoretical specific energy (2600 Wh kg^−1^) and high natural abundance, low cost, and environmentally benign nature of elemental S material.^[^ ^1^, ^2^ ^]^ However, many challenges of LSBs have hindered the implementation of this battery system in practical applications.^[^ ^3^, ^4^, ^5^ ^]^ The progress in fundamental understanding and the improvements in multiple aspects of LSBs achieved in past decades^[^ ^6^, ^7^, ^8^, ^9^ ^]^ have indicated that the most prominent challenges to LSBs are correlated to the stabilization of the Li metal anode and the S redox chemistry, the insulating nature of S and Li_2_S (the final discharge product), and the severe shuttling of polysulfides (PSs) causing self‐discharge of LSBs.^[^ ^4^, ^5^ ^]^ The root of many of these issues is the dissolution of the soluble and reactive PS intermediate species that migrate from the S cathode and deposit onto the anode surface of the cell leading to continuous parasitic side reactions, loss of active materials, increase in cell impedance and polarization, and eventually decrease of the electrochemical performance of LSBs.^[^ ^10^ ^]^ Extensive efforts in nearly all aspects of LSBs have been made in recent years to mitigate PS dissolution and its negative effects.

There have been many strategies for modifying S cathode architecture to improve electrode conductivity, enhance PS conversion and reduce PS dissolution^[^ ^11^, ^12^, ^13^, ^14^ ^]^; imbedding redox mediation catalysts into S cathode to lower the thermodynamic energy barrier to efficiently convert soluble long‐chain PSs to short‐chain PSs^[^ ^15^, ^16^ ^]^; modifying or coating the separator to retain PS species and inhibit PS shuttling to the anode^[^ ^17^, ^18^, ^19^ ^]^; formulating electrolytes with inclusion of additives to improve S electrode wettability,^[^ ^20^, ^21^ ^]^ enhance PSs conversion kinetics,^[^ ^22^, ^23^ ^]^ and control PSs amount or suppress PS dissolution into electrolyte.^[^ ^24^, ^25^, ^26^ ^]^ It is known the electrolyte plays a significantly critical role in controlling PS dissolution and conversion besides stabilizing Li metal anode, then affecting LSB performance. In general, the liquid electrolytes for LSBs reported in literature can be grouped into three main categories: 1) highly solvating electrolytes (HSEs), 2) moderately solvating electrolytes (MSEs), and 3) sparingly solvating electrolytes (SSEs).^[^ ^24^, ^27^ ^]^ HSEs, such as the conventional ether‐based electrolyte of 1 m Li bis(trifluoromethanesulfonyl)imide (LiTFSI) in 1,2‐dimethoxyethane and 1,3‐dioxolane (DME/DOL, 1:1 by vol.) with additive Li LiNO_3_, emphasize on promoting PSs solubility and fast Li‐S reaction kinetics with less electrolyte utilization, but in turn promoting PSs shuttling and causing poor Li metal stability and short cycle life.^[^ ^28^ ^]^ On the other end of the spectrum, SSEs, such as high‐concentration electrolytes (HCEs), localized high‐concentration electrolytes (LHCEs)^[^ ^29^, ^30^, ^31^ ^]^ and electrolytes with large molecule solvents or ionic liquids (ILs),^[^ ^32^, ^33^, ^34^ ^]^ are designed to significantly reduce LiPSs solubility to inhibit LiPSs shuttling and subsequent parasitic reactions, but they reduce the overall cell capacity and cyclability due to the high viscosity or low ion conductivity, and poor conversion of PSs.^[^ ^17^ ^]^ Very recently, MSEs have been reported to balance the advantages and issues existing in HSEs and SSEs, anticipating to be a medium of controlled PS solubility to inhibit the PSs shuttling effect without sacrificing the high S utilization and fast Li–S reaction kinetics required to enable a stable long‐term cyclability of LSBs with a high discharge capacity.^[^ ^27^ ^]^ However, how to design a well‐balanced MSE remains unclear.

## 2 Results and Discussion

### 2.1 Designing MSEs with Multiple Solvent Systems to Achieve Advanced LSB Performance

In conventional HSEs and SSEs for LSBs, binary solvent systems are normally used, with a highly solvating solvent and a moderately solvating solvent for HSEs while a highly solvating solvent or a moderately solvating solvent and a sparingly solvating solvent for SSEs. The combination of a highly solvating solvent and a moderately solvating solvent is hard to reduce the PS solubility to a moderate level, for instance, the DME and DOL mixtures used in conventional LSB electrolytes which always result in high solubility of LiPSs. On the other hand, the mixture of a highly solvating solvent or a moderately solvating solvent and a sparingly solvating solvent can be tuned to adjust the PS solubility to a moderate level, however, the pre‐added Li salt in this solvent mixture to make the electrolyte would largely influence the solubility of the formed LiPS species and later the conducting Li salt itself, thus leading to large variation in solubility of Li salt and LiPSs during repeated charge and discharge cycles of the LSB. To design feasible MSEs for LSBs, a multiple solvent system containing a highly solvating solvent, a moderately solvating solvent and a sparingly solvating solvent is selected in this work. The LiPS solubility of this electrolyte system can be tuned to overcome both the low solubility problem (encountered in the electrolyte with moderately solvating solvent and sparingly solvating solvent) and the high solubility problem (fast dissolving and shuttling of LiPSs with highly solvating solvent).

The capability of a solvent to solvate a cation or a Lewis acid species is related to its dielectric constant (*ɛ*) and/or donor number (DN). A higher ɛ or DN indicates the solvent can more easily solvate or coordinate more cations. Solvents with low DN are found to have poor ability to solvate Li^+^ and stabilize PSs with higher charge density.^[^ ^29^, ^35^ ^]^ Besides the solubility of LiPSs in electrolyte, the reactivity or compatibility of each solvent in the electrolyte against Li metal and LiPS species should also be considered. The reactivity of the solvent against Li metal can be first screened by the lowest unoccupied molecular orbital (LUMO) energy of the solvent and further evaluated by the average Li Coulombic efficiency (CE) of the solvent‐based electrolyte in Li||copper (Cu) cells, while the reactivity of the solvent against LiPSs can be judged by the local softness value of the solvent via density function theory (DFT) calculation,^[^ ^20^, ^21^, ^22^, ^36^ ^]^ as the reactions between LiPSs and solvents in LSBs are usually considered as nucleophilic attack.^[^ ^23^ ^]^ Empirically, a solvent with a high LUMO energy and a low local softness value would suggest good stability against Li metal and LiPSs, respectively.

**Figure 1:**
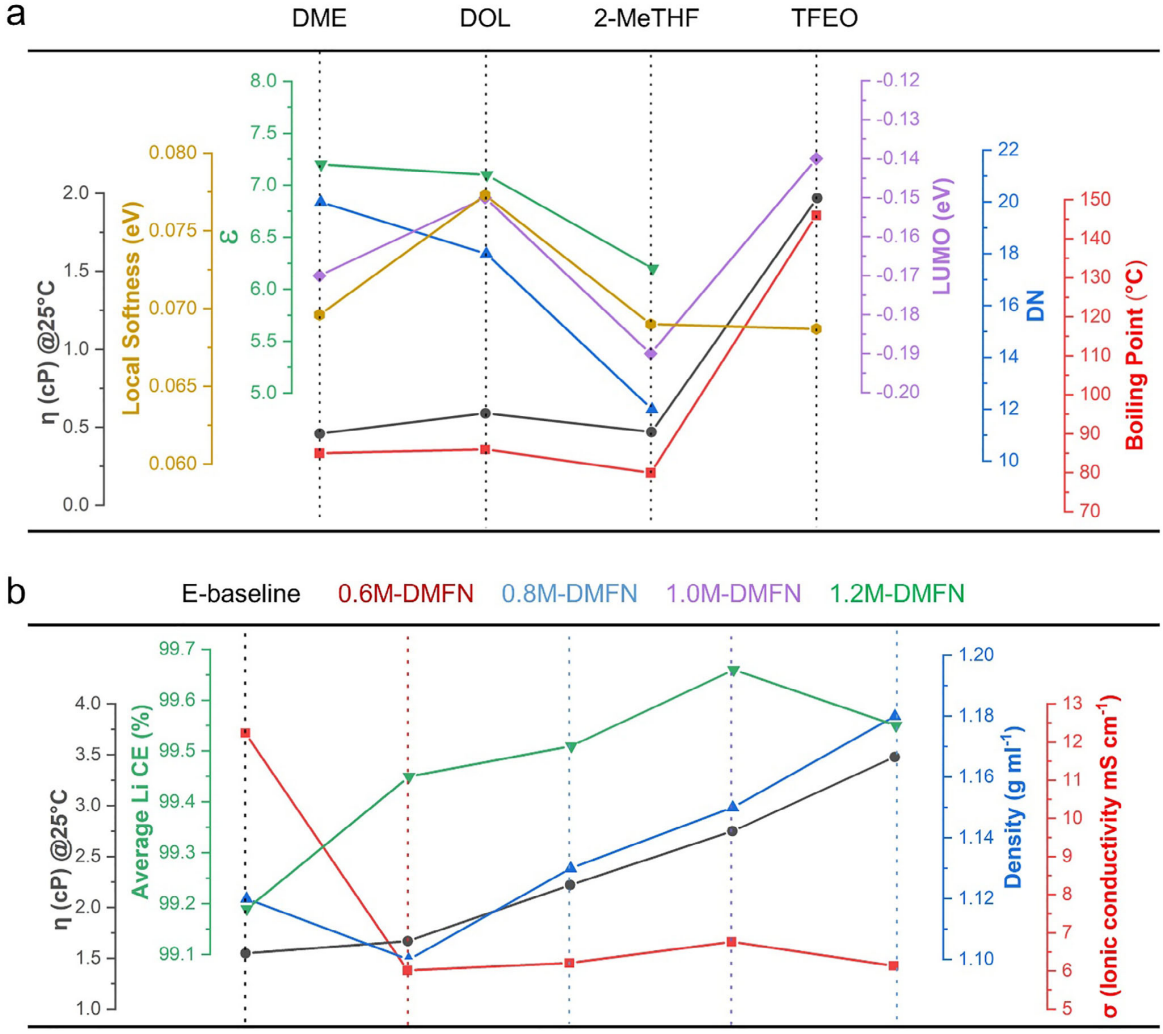
Physical and electric properties of selected solvents and studied electrolytes. a) Selected solvents of DME, DOL, 2‐MeTHF, and TFEO to make electrolytes. b) E‐baseline and DMFN electrolytes.

In this work, DME, 2‐methyltetrahydrofuran (2‐MeTHF) and tris(2,2,2‐trifluoroethyl)orthoformate (TFEO) were selected as the model solvents to prepare MSEs due to their appropriate ɛ and DN values (for high, moderate, and sparing solubilities of LiPSs), low local softness (indicating good stability against LiPSs), and relatively high LUMO energy (suggesting good compatibility with Li metal).^[^ ^35^, ^37^, ^38^, ^39^, ^40^, ^41^ ^]^ The physical properties of the four solvents are shown in **Figure** **1**a. It is seen that 2‐MeTHF has lower DN (12) and ɛ (6.2) than DME (DN 20, ɛ 7.2) and DOL (DN 18, ɛ 7.1), which makes it have less capability of coordinating Li ions (Li^+^) than DME and DOL. In addition, TFEO, a hydrofluoroether, should have much lower DN and ɛ than regular ethers have, indicating it is unable to coordinate with Li^+^ and consequently has low to no solubility of LiPSs. These approximations can be verified by the solubility of Li_2_S_6_ in DME, DOL, 2‐MeTHF, and TFEO shown in Figure S1a (Supporting Information), indicating the solvating capability of Li_2_S_6_ from high to moderate and to sparing degree is DME > DOL > 2‐MeTHF > TFEO. The white TFEO solution shows no reaction between the S_8_ and Li_2_S powders in TFEO, confirming that TFEO acts as a non‐coordinating solvent for LiPSs and there is no reaction with or dissolution of Li_2_S_6_. On the other hand, the calculated local softness values for oxygen (O) atom on C─O bond in the four solvents have the order from high to low as DOL (0.0773 eV^−1^) > DME (0.0696 eV^−1^) ≈2‐MeTHF (0.0690 eV^−1^) ≈TFEO (0.0687 eV^−1^) (Figure 1a), suggesting the lower reactivity of TFEO, 2‐MeTHF and DME than that of DOL in the reactions between PSs and solvent. In addition, the moderately solvating solvent, 2‐MeTHF, has been reported to improve the cyclability and reversibility of the Li metal anode (LMA).^[^ ^37^ ^]^ Furthermore, TFEO, has been reported to function as a non‐coordinating diluent that contributes to the formation of a F‐rich solid electrolyte interphase (SEI), enhance the compatibility with LMA, and improve the cell stability and safety.^[^ ^38^, ^39^, ^40^, ^41^ ^]^ The higher boiling point of TFEO may also enhance the thermal stability of the electrolytes at elevated temperatures and can be a factor for improving the stability and thermal safety of LSBs.

DME, 2‐MeTHF and TFEO were blended in a 2:1:1 volume ratio with 2 wt.% LiNO_3_ (a typical additive to passivate LMA and suppress PS shuttling effect).^[^ ^42^ ^]^ LiTFSI salt concentration in the range of 0.6–1.2 m was investigated in this work, which from hereafter are referred to be the “DMFN” electrolytes. In comparison, the conventional electrolyte of 1 m LiTFSI in DME/DOL at 1:1 volume ratio with 2 wt.% LiNO_3_ was also blended and named as E‐baseline. The basic electrochemical and physical properties of the electrolytes were investigated and summarized in Figure 1b and Figure S2 (Supporting Information). The ionic conductivities of the DMFN electrolytes are about half of that of E‐baseline, mainly because of the low solvating capacity of 2‐MeTHF and TFEO, which is desired for lowering the LiPSs solubility. However, the ionic conductivity of 6–7 mS cm^−1^ at 25 °C for DMFN electrolytes is comparable to most conventional carbonated electrolytes used in Li‐ion batteries. Among the four DMFN electrolytes with salt concentrations from 0.6 to 1.2 m, the maximum conductivity of 6.77 mS cm^−1^ is obtained at 1.0 m. The viscosities of E‐baseline and 0.6M‐DMFN are nearly overlap, but the viscosities of other DMFN electrolytes increase with increasing the salt concentration although their ionic conductivities are nearly the same (see Figure S2a, Supporting Information).

The stability of E‐baseline and DMFN (0.6– 1.2 m) electrolytes with Li metal was investigated using the modified Aurbach method^[^ ^43^ ^]^ in Li||Cu cells (see Figure 1b; Figure S3, Supporting Information). The maximum CE of 99.66% was obtained for 1.0M‐DMFN. This is much higher than 99.19% obtained for E‐baseline. The improved Li CE in DMFN electrolytes over E‐baseline can be attributed to the 2‐MeTHF and TFEO co‐solvents which have been previously reported to improve stability with Li metal.^[^ ^38^, ^39^, ^40^, ^41^ ^]^

**Figure 2:**
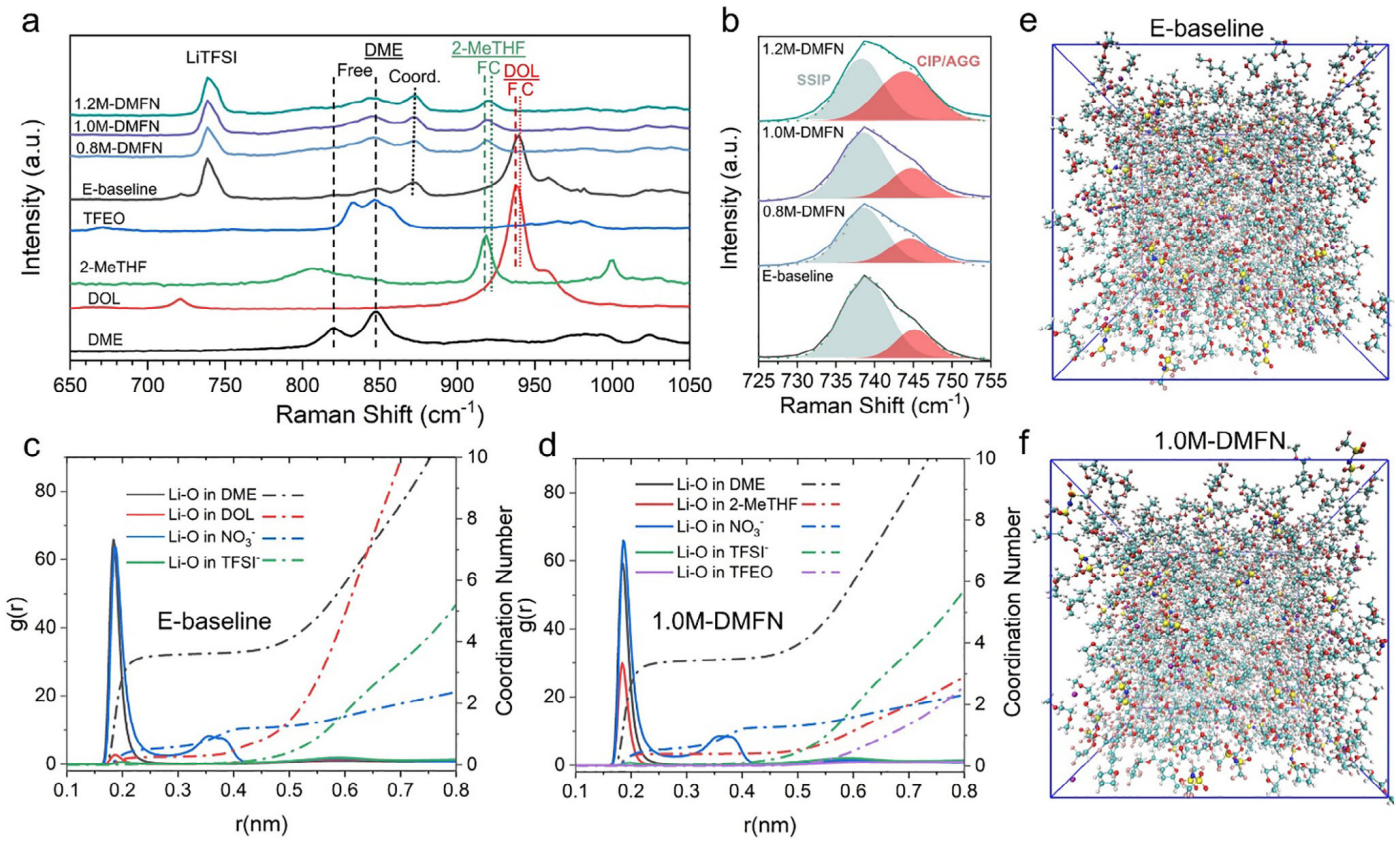
Coordination and solvation structure of selected electrolytes. a,b) Raman spectra of solvents and electrolytes (E‐Baseline and DMFNs). c,d) Radical distribution function curves and coordination numbers of Li‐O bonds in solvents and anion pairs calculated from Classical molecular dynamics simulation for E‐baseline and 1.0M‐DMFN. e,f) Solvation structures of E‐baseline and 1.0M‐DMFN.

To understand the Li^+^ coordination environment of the DMFN electrolytes, Raman analysis was performed for pure solvents as well as E‐baseline and DMFN electrolytes (**Figure** **2**a). Raman spectra indicate that DME is the primary coordinating solvent in both E‐baseline and DMFN electrolytes, with the weakly solvating DOL and 2‐MeTHF co‐solvents showing less significant peak reduction and shifting in the peaks attributed to the of non‐coordinating free solvent present in the electrolyte (Figure S4, Supporting Information) and TFEO showing no coordination of Li^+^. The peak analysis of the coordination environment of LiTFSI (Figure 2b) reveals that E‐baseline has the highest degree of solvent separated ion pairs (SSIPs), even when compared to the DMFN with lower LiTFSI concentration. The intensity of the peak for contact ion pairs (CIPs) and aggregates (AGGs) increases with increasing LiTFSI concentration of the DMFN electrolytes (Figure 2b; Figure S5, Supporting Information). The addition of TFEO, as a diluent, incorporates LHCE‐like properties in the DMFN electrolytes, increasing the ratio of CIPs/AGGs in the low concentration DMFN electrolytes.

Classical molecular dynamics (CMD) was used to simulate the molecular structures of electrolytes as described in the Supporting Information. The refractive indices of solvents are listed in Table S1 (Supporting Information) and the molecular structures of the solvents and salt anions are shown in Figure S6a (Supporting Information), and the simulated snapshots of the different electrolytes are represented in Figure 2e,f and Figure S6b–d (Supporting Information), together with the corresponding radial distribution function (RDF) curves of the oxygen (O) atoms in salt anions and solvents to Li^+^ for E‐baseline and the representative 1.0M‐DMFN electrolyte, shown in Figure 2c,d. The solvation environment of Li^+^ in E‐baseline is predominantly coordinated with O_DME_ and to a lesser extent O_DOL_. The coordination of Li‐O_TFSI_ is nearly zero in the first solvating shell, indicating that the salt is nearly fully solvated in the electrolyte. The solvation structure of the DMFN electrolyte series is found to be identical, regardless of the concentration of LiTFSI (Figure 2c,d; Figure S6e–g, Supporting Information). The Li^+^ is again predominately coordinated with O_DME_ and an observed more prominent Li^+^ coordination with O_2‐MeTHF_ compared to O_DOL_ in E‐baseline. There is no observed coordination for Li‐O_TFEO_ confirming that TFEO is effective as a non‐coordinating diluent in the DMFN electrolyte system and does not interact with Li^+^. These results align well with the indications from the DN and local softness values as shown in Figure 1a, as well as the Raman results, confirming that 2‐MeTHF functions as a weakly solvating solvent and TFEO is a non‐coordinating diluent in the DMFN electrolytes.

As shown in Figure S7a (Supporting Information), there is a significant difference in the colors of the Li_2_S_6_ solutions between E‐baseline and DMFN electrolytes after stirring at 55 °C. The dark brown color of the LiPS‐containing E‐baseline indicates a high concentration of dissolved LiPSs, while the LiPS‐containing DMFN electrolytes maintain a lighter green‐brown color indicating an overall lower concentration of dissolved LiPSs. The concentration of LiTFSI also affects the degree of LiPS solubility as the color of the Li_2_S_6_ solution is lighter with increasing the LiTFSI concentration from 0.6 to 1.2 M in the DMFN electrolytes. While this qualitative observation confirms that the designed MSEs have lower solubility of LiPSs, UV–vis spectroscopy was further utilized for a more quantitative estimation. The UV–vis spectra in Figure S7b (Supporting Information) confirm that all DMFN electrolytes have a noticeably lower intensity for S related peaks in the 1.5 m Li_2_S_6_ solutions indicating the suppression of long‐chain PSs in the DMFN.

The elemental analysis was conducted to measure the total S content in the five electrolytes. As shown in Table S2 (Supporting Information), the total S content in the four DMFN electrolytes is lower than that in E‐baseline; however, it is impossible to get the quantitative concentration of Li_2_S_6_ in these electrolytes by using the elemental analysis results. In another effort, the maximum concentrations of Li_2_S_6_ in the two solvent systems for the baseline and DMFN electrolytes were analyzed and calculated to be 0.83 m for DME‐DOL (2:1 by vol) and 0.38 m for DME‐2MeTHF‐TFEO (2:1:1 by vol), as shown in Tables S3 and S4 (Supporting Information), suggesting that the LiPS concentration in the DMFN electrolytes would fall into the moderate concentration range.

Electrode soaking results shown in Figure S8 (Supporting Information) also demonstrates that E‐Baseline could slightly dissolve S_8_ in the pristine S electrode in only 3 days but the 1M‐DMFN with S electrode showed no color change over 7 months. The interactions between the LiPSs with the different electrolytes were studied by CMD simulations for Li_2_S_6_ as the representative LiPS in the electrolytes, and the details are reported in the Supplementary Section of Coordination Environment of MSEs and Figures S9–S13 (Supporting Information).

### 2.2 Electrochemical Performance of DMFN Electrolytes

**Figure 3:**
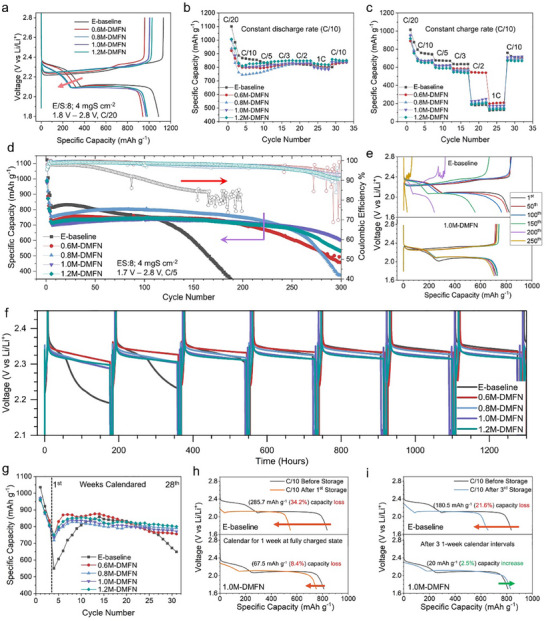
Electrochemical evaluation and calendar aging behavior of E‐baseline and DMFN electrolytes in Li||S cells. a) Voltage profiles of the initial formation cycle at C/20 rate. b,c) Rate capability evaluation of Li||S cells for charge and discharge, respectively. d) Cycling performance of LSBs using E‐baseline and DMFN electrolytes at C/5 rate. e) Voltage profiles of the selected cycles for E‐baseline and 1.0M‐DMFN electrolytes cycled at C/5 rate. All cells were cycled at 25 °C and LSBs were first cycled with 2 formation cycles at C/20 between 1.8 to 2.8 V followed by C/10 or C/5. f) Voltage profiles of self‐discharge behavior over the first 7 one‐week intervals. g) Cycling profiles of discharge capacity after resting for one‐week over the total period of 28 consecutive one‐week intervals. h,i) Comparison of discharge voltage profiles of LSBs with E‐baseline and 1.0M‐DMFN electrolyte after the 1st and 3rd one‐week resting intervals compared to the C/10 cycling before resting, respectively.

The electrochemical performance of E‐baseline and DMFN electrolytes in LSBs was investigated in coin cells using a S/carbon composite electrode with an areal capacity loading of 4 mAh cm^−2^ and an electrolyte/sulfur (E/S) ratio of 8 µL mg^−1^‐S for the electrolyte. The charge and discharge voltage curves of the first formation cycle in **Figure** **3**a reveal that in the first discharge plateau, which is associated with the formation of longer chain PSs (Li_2_S_6‐8_), there is a noticeable decrease in the specific capacity from E‐baseline to the DMFN electrolytes and the specific capacity decreases with increasing salt concentration of the DMFN electrolytes. The moderately solvating DMFN electrolytes reduce the amount of long‐chain PSs in the electrolytes, which in turn reduces the overall capacity of the LSBs. The effects of the charge and discharge rates on the cell performance of LSBs were evaluated and compared between E‐baseline and DMFN electrolytes (Figure 3b,c). As shown in Figure 3b, the charge rate is not a limiting factor to enable higher rate performance of the LSB. The E‐baseline and DMFN electrolytes had an initial difference in capacity during the first few cycles at the initial C/10 rate but quickly began to overlap during the C/5 charge rate. There is no noticeable drop in capacity with increasing C‐rate until charging at 1C with the capacities for all electrolytes remaining overlapped. However, the discharge rate is found to be a more significant factor in LSB performance, as exhibited in Figure 3c. There was a consistent decrease in specific capacity with each increasing discharge rate and nearly all electrolytes showed a sudden capacity drop when the discharge rate was increased to C/2, except for the 0.6M‐DMFN electrolyte which was able to give a reasonable capacity until the discharge rate was increased to 1C. The charge‐discharge voltage profiles of LSBs with E‐baseline and 0.6M‐DMFN electrolytes at selected discharging rates are compared in Figure S14 (Supporting Information). The rate performance results indicate that the conversion of the long‐chain PSs to the short‐chain PSs is the rate‐limiting step during discharge that creates a large overpotential in the cell and causes the LSB to prematurely reach the cut‐off voltage specially at higher C rates at and above C/2, even at C/3 the dip at the start of second plateau reflects the kinetics issue for the formation of short‐chain PSs in E‐baseline. Though the four DMFN electrolytes have lower ionic conductivity and higher viscosity than E‐baseline (Figure 1b), which make the LSBs give lower discharge capacity in the initial cycles than E‐baseline, they do have higher average Li CEs (Figure 1b) and much lower LiPS solubility (Figure S7a, Supporting Information) than E‐baseline, which cause less side reactions between electrolyte and Li metal and much less loss of S active material from cathode and largely reduced crosstalk of LiPSs from cathode to anode. In addition, the ionic conductivities of the DMFN electrolytes are still moderately high (≈6–7 mS cm^−1^), therefore the combination of these factors makes the DMFN electrolytes exhibit similar charge and discharge rate capabilities to E‐baseline when the discharge rate is at or below C/3.

The electrochemical performance of the LSBs with E‐baseline and DMFN electrolytes at a constant charge/discharge rate of C/5 over 300 cycles was further evaluated (Figure 3d). While E‐baseline had a higher initial capacity because of low viscosity and more wettability of cathodes, the DMFN electrolytes showed significantly improved cycling stability and CE, with the 0.8M‐DMFN exhibiting the highest specific capacity among the DMFN electrolytes and the 1.0M‐DMFN electrolyte the best cycling stability having a high‐capacity retention of 81.3% (604 mAh g^−1^) after 300 cycles. The charge/discharge voltage curves of selected cycles at C/5 are compared in Figure 3e and Figure S15 (Supporting Information). Over a period of 250 cycles, the voltage profiles of E‐baseline containing LSBs showed significant growth in cell polarization, overcharging of the cells, and rapid capacity degradation after 100 cycles highlighting the high reactivity of E‐baseline with both S and Li electrodes. In comparison, all DMFN LSBs exhibited low polarization over 250 cycles with the 1.0M‐DMFN containing cells showing the least (or no significant) polarization or decrease in capacity between the 1st and 250th cycles at C/5 rate. The cycling performance and voltage profiles of LSBs using these electrolytes at a slower rate (C/10) are compared in Figures S16 and S17 (Supporting Information), respectively. The overall electrochemical performances of E‐baseline and DMFN electrolytes demonstrate that the DMFN electrolytes significantly enhance the cell stability and cyclability, with the observations that 0.8M‐DMFN generally enables the highest capacity while at C/5 the 1.0M‐DMFN has the best cycling stability suggesting the LiTFSI concentration for optimal capacity and stability is between 0.8 and 1.0 m. Furthermore, the 0.8M‐DMFN and E‐baseline electrolytes were evaluated in 1‐Ah pouch cells and the preliminary cycling results shown in Figure S18 (Supporting Information) also indicate the 0.8M‐DMFN shows better cycling stability than E‐baseline where the latter exhibits large overpotential after 40 cycles because the extra charging capacity from unwanted side reactions leads to low Coulombic efficiency ultimately to cell death.

In addition to cycling stability, calendar aging stability is another important metric for LSBs to determine the stability of the S and Li electrodes against the electrolyte during long‐term storage. The S electrode is inherently more reactive with the electrolyte compared to the conventional Li‐ion intercalation cathodes due to the ability to continuously dissolve S and LiPSs, be suspended in the electrolyte, and migrate to and react with the LMA leading to irreversible loss of active materials, depletion of electrolyte and insulation of Li anode. This phenomenon is prevalent in E‐baseline due to its high solubility and reactivity with S and LiPSs.^[^ ^24^ ^]^ It is well known that self‐discharge is a big issue in LSBs with liquid electrolytes, resulting in poor calendar life of LSBs.^[^ ^44^ ^]^ Therefore, to test the self‐discharge of LSBs with E‐baseline and DMFN electrolytes at the charged state over 1‐week intervals for a period of 28 weeks can give us the calendaring stability of LSBs with these electrolytes.

Figure 3f,g shows the behavior of the LSBs with E‐baseline and DMFN electrolytes in terms of the voltage profiles and discharge capacity during the calendaring and self‐discharge tests, respectively. After two formation cycles and one regular charge/discharge at C/10 as a reference cycle, there is an apparent difference between the LSBs in E‐baseline and all four DMFN electrolytes. The E‐baseline containing cell had a significant voltage decay after 50 h into the first calendaring period and the voltage decreased to 2.2 V after the first week with a large drop in capacity (34.2%) (Figure 3f,h). The E‐baseline containing cell took multiple weeks of cycling and calendaring periods for the voltage profile to recover and stabilize, and it took 12 one‐week intervals to recover its discharge capacity to the initial reference cycle, however, the E‐baseline cell was unable to maintain its recovered capacity for more than 4 intervals before undergoing capacity fading for the remaining intervals (Figure 3g). In contrast, the cells with DMFN electrolytes maintained significantly better voltage stability and self‐discharge suppression during the calendaring periods (Figure 3f). All DMFN cells had a considerably less drop in their discharge capacities of ≈8% for all concentrations after the first calendaring period and increased their discharge capacity per cycle after just 3 weeks. All DMFN electrolytes had a high level of voltage and capacity stability over the 28 one‐week intervals (Figure 3g).

The discharge voltage curves after the aging period accentuate the enhanced stability of the DMFN electrolytes, with 1.0M‐DMFN chosen as the representative electrolyte, compared to E‐baseline (Figure 3h,i). The apparent loss of the plateau in 2.4–2.1 V during discharge, which corresponds to the voltage plateau of transferring Li_2_S_8_ to Li_2_S_6_ in a typical discharge process, in the E‐baseline cell can attribute to the high solubility of Li_2_S_8_ in E‐baseline. It is concluded that the suppression of LiPSs solubility and the more robust passivation layer on the S and Li electrodes, observed in the DMFN electrolytes, more effectively mitigate the S/LiPSs shuttling effect so that it suppresses the self‐discharge of the LSBs during storage or calendar aging and improves the long‐term health of the LSBs.^[^ ^45^, ^46^ ^]^

### 2.3 Quantification of S and Metallic Li (Li^0^) Inventory after Cycling

**Figure 4:**
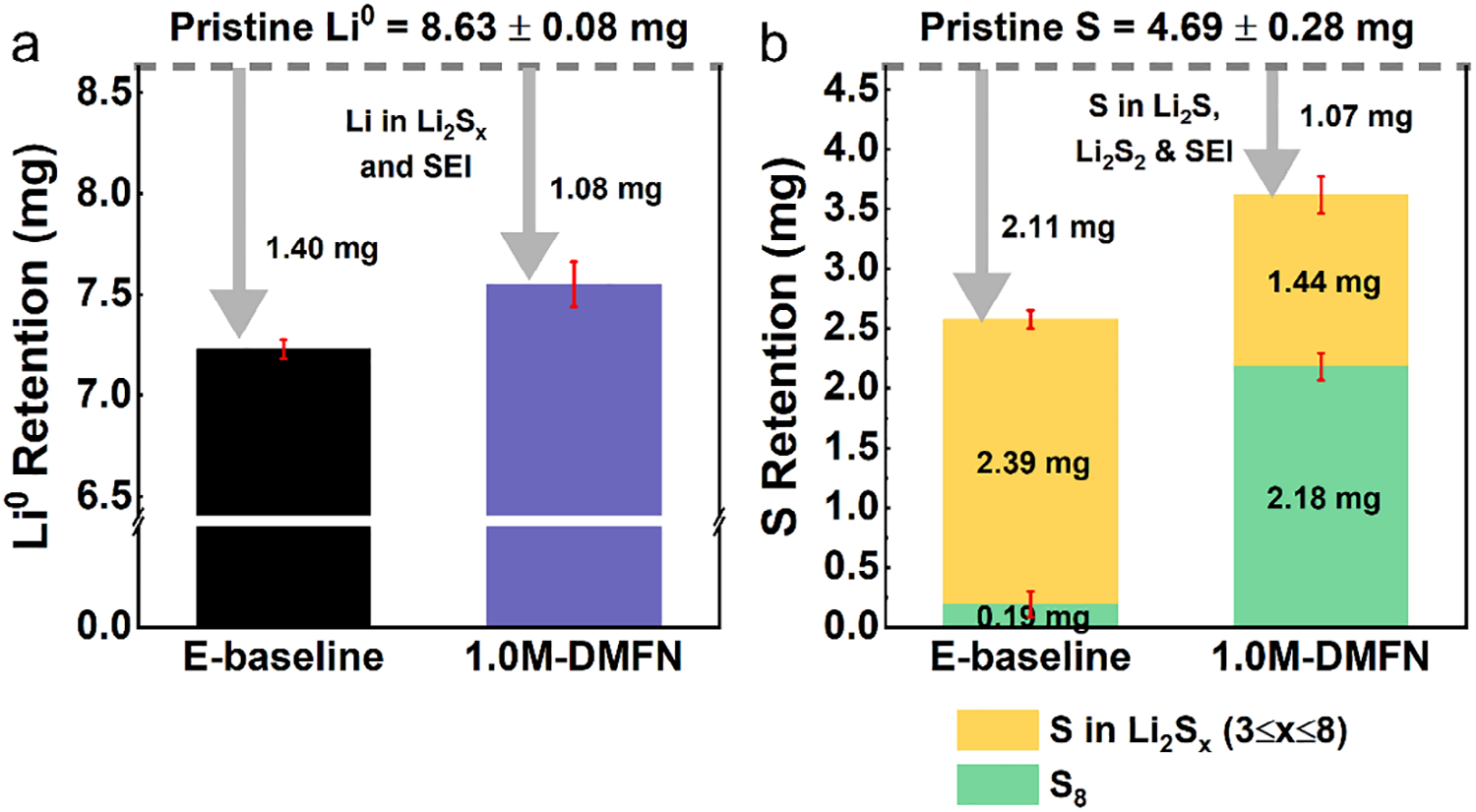
Post‐mortem inventory retention quantification after 100 cycles at C/5. a) Li^0^ and b) S cycled electrodes.

Understanding the degree and mechanism of S and Li^0^ utilization is crucial for designing compatible electrolytes for sustainable interactivity between the cathode, anode, and electrolyte in LSBs and determining if the design of the DMFN MSEs is on the right direction. To gain insight on the utilization, speciation, and retention of S, LiPSs, and Li^0^ in LSBs after cycling in both E‐baseline and 1.0M‐DMFN, the HPLC, UV–vis, GC‐MS (HUGs) method developed by Meng and colleagues was employed.^[^ ^47^ ^]^ As shown in **Figure** **4**, retention for Li^0^ after 100 cycles reveals that 1.0M‐DMFN cycled Li^0^ has a lower amount of irreversible Li^0^ consumption that is assigned to the side reactions in the formation of the SEI, Li_2_S, and Li_2_S_2_ and has improved the overall Li^0^ retention, corroborating the improved Li° conditions observed in the post‐mortem characterization results. More significantly, the disparity in the S retention and speciation of the cycled S electrodes is more apparent between the two electrolytes. When cycled in E‐baseline, the amount of irreversible loss of active S material from the electrode is almost double of S loss in 1.0M‐DMFN at 45.3% and 23.1%, respectively. The remaining S from the E‐baseline cells was determined to be almost entirely in LiPSs (3 ≤ x ≤ 8) measured to be 92.6% and 7.4% in S_8_ phase of reversible S remaining in the cells, while the 1.0M‐DMFN cells were measured to have 36.7% and 63.3% in the LiPSs and S_8_ phases, respectively. The designed MSE DMFN series retains a significantly improved amount of active S in the cycled electrodes with most of the active S remaining in the S_8_ phase while minimizing LiPSs speciation in the cells after 100 cycles. On the other hand, when cycled in E‐baseline, an HSE, the S was primarily converted to LiPSs and continuously dissolved in the electrolyte leading to continuous side reactions and accelerated loss of S from the electrode leading to poor CE and accelerated cell degradation.

### 2.4 Post‐Mortem Analyses of Cycled Li and S Electrodes

**Figure 5:**
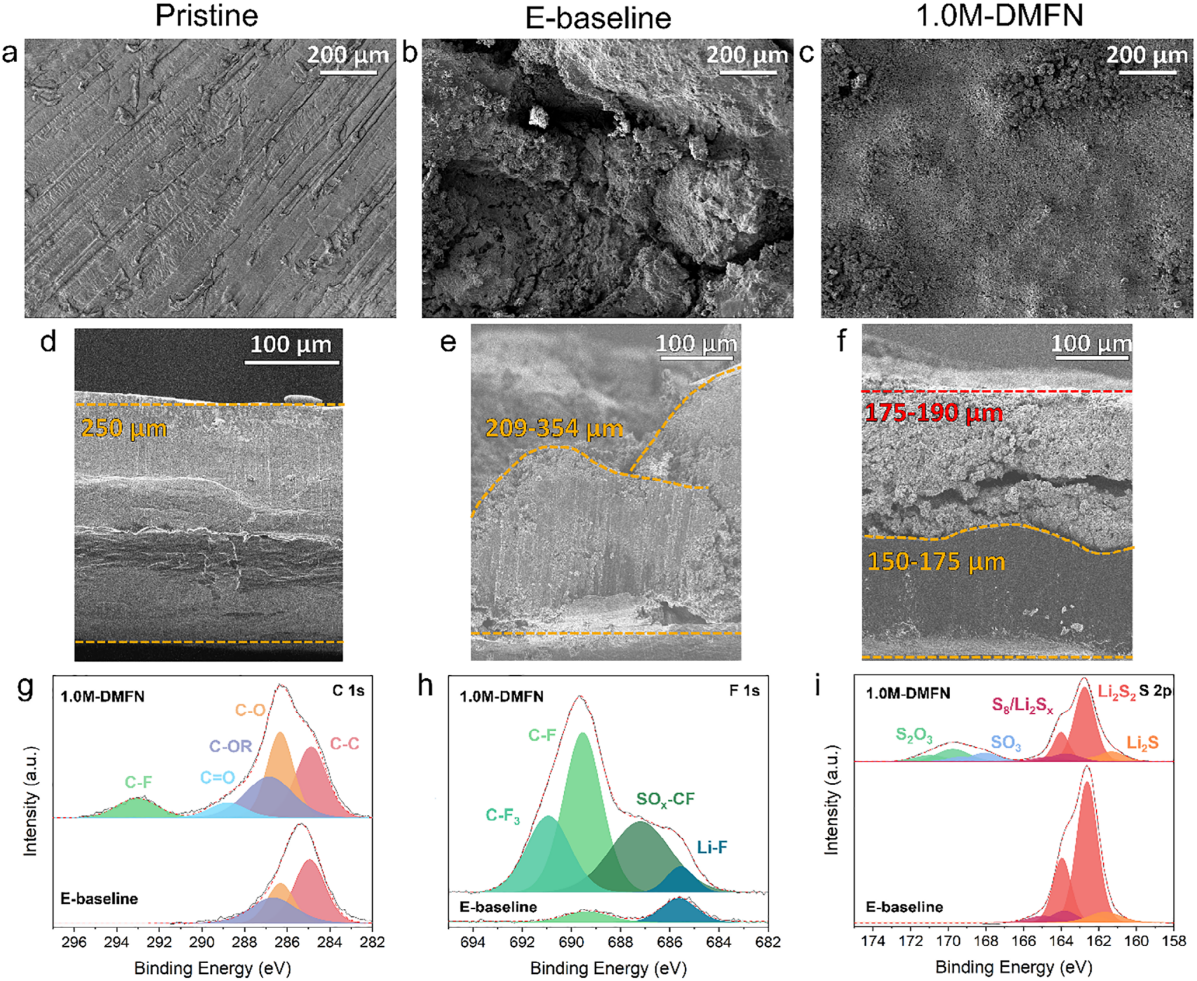
Post‐mortem characterization of LMAs after 300 cycles at C/5. a–c) Top‐down SEM images of pristine and cycled LMAs from E‐baseline and 1.0M‐DMFN cycled cells, respectively. d–f) Cross‐section SEM images of pristine and cycled LMAs in E‐baseline and 1.0M‐DMFN electrolytes, respectively. g–i) XPS analysis of C 1s, F 1s, and S2 p spectra from cycled LMAs in E‐baseline and 1.0M‐DMFN cells.

After 300 cycles at C/5, the LMAs and S cathodes from the LSBs were retrieved and characterized. Optical photographs of the cycled LMAs presented in Figure S19 (Supporting Information) show the LMA from the E‐baseline containing LSB suffered significant corrosion and pitting throughout the LMA, but the corrosion of the LMAs from the DMFN LSBs was less severe and the severity decreased with increasing the salt concentration in the electrolyte. The LMA from the 1.0M‐DMFN cell showed the least amount of noticeable corrosion and pitting, corroborating with the best Li CE observed in Figure S3 (Supporting Information) and Figure 1b and the best long‐term cycling stability shown in Figure 3d, with the improved condition of the LMA. Therefore, the pristine and cycled LMAs from the E‐baseline and 1.0M‐DMFN containing cells were further characterized using scanning electron microscopy (SEM) and X‐ray photoelectron spectroscopy (XPS). As shown in **Figure** **5**a–f for the images of the top‐down and cross‐section, there is a stark difference in the morphologies of the LMAs cycled in E‐baseline and 1.0M‐DMFN electrolytes. The LMA from the E‐baseline cell had a highly rough and non‐flat surface and suffered from deep pitting throughout the LMA (Figure 5b), while the cycled LMA with 1.0M‐DMFN had a homogeneously dense microstructure morphology throughout the entirety of the surface of the LMA (Figure 5c). Higher magnification SEM images further highlight the difference in the microstructure of the plated Li in the E‐baseline and 1.0M‐DMFN LSBs (Figure S20, Supporting Information). The cross‐sectional SEM images also reveal that the Li metal cycled in the E‐baseline cell was significantly more corroded with a thick, highly reacted, Li layer ranging 209–350 µm that penetrated nearly the entire 250 µm Li metal disc (Figure 5e). The cross‐section of the LMA cycled in 1.0M‐DMFN revealed two distinctive Li layers: a homogeneous and dense Li reaction layer that was 175–190 µm thick and a 150–175 µm thick layer of unreacted Li (Figure 5f), where the cracking was probably caused during the sample preparation. The higher Li CE and improved long‐term cycling stability of the LSBs with 1.0M‐DMFN electrolyte can be attributed to the suppressed LiPS shuttle effect which in turn improves the morphology of deposited LMA and minimizes its corrosion.

The C 1s XPS spectra (Figure 5g) show the presence of a richer organic phase on the LMA from the 1.0M‐DMFN cell with an additional C‐F peak that is not observed for LMA from the E‐baseline cell. The F 1s spectra (Figure 5h) further reveal the significantly increased variety and intensity of fluorinated species in the SEI on LMA from the 1.0M‐DMFN due to the inclusion of TFEO that has been reported to form a more homogeneous and F‐rich SEI that enhanced the protection of and stability with Li metal during cycling.^[^ ^40^, ^48^ ^]^ A weaker intensity for LiPS species was measured on the surface of Li metal cycled in 1.0M‐DMFN in the S 2p spectra along with a stronger intensity for peaks related to decomposed TFSI^−^ byproducts that form a more robust, anion‐rich SEI layer (Figure 5i). The lower intensity of LiPS species on the LMA from the 1.0M‐DMFN cell further corroborates a reduction in LiPS dissolution and shuttling in the DMFN electrolytes in the LSBs. The higher intensity N‐S peak measured in the DMFN cycled LMA in the N 1s spectra (Figure S21, Supporting Information) corroborates a more inorganic SEI of decomposed TFSI^−^ byproducts that further protects the LMA from corrosion while cycling. The observed physical condition and chemical environment of the cycled LMA in 1.0M‐DMFN demonstrates that the DMFN electrolyte significantly has enhanced compatibility with the LMA and reduced LiPS migration and parasitic reactions inhibiting the PS shuttle effect, thus mitigating the corrosion and inactivity of the LMA.

For the S electrodes, SEM imaging shows the pristine S electrode has large primary particles of ≈50 µm throughout the surface of the electrode (Figure S22a, Supporting Information). After 300 cycles, the morphology of the S electrode in E‐baseline has significant S particle delamination and agglomeration across the electrode surface (Figure S22b, Supporting Information). However, the S electrode cycled in 1.0M‐DMFN has a homogeneous distribution of dense S particle structure throughout the surface of the electrode (Figure S22c, Supporting Information). The corresponding energy dispersive X‐ray (EDX) mapping of the surface of the pristine S electrode shows large sections of bare C (blue) and S (yellow) (Figure S23a, Supporting Information), the E‐baseline cycled S electrode also shows large sections of C but relatively homogeneous S distribution (Figure S23b, Supporting Information), while the DMFN cycled electrode maintains a more homogeneous distribution of selected elements (C, O, F, and S) and more prominent S atom% throughout the electrode (Figure S23c, Supporting Information). Plasma focused ion beam (PFIB)‐SEM imaging of the S electrodes further reveals a collapse of the S electrode architecture and agglomeration of S particles in the bulk of the electrode after cycling in E‐baseline (Figure S23e, Supporting Information), when compared to the structure of the pristine S electrode (Figure S23d, Supporting Information) and the S electrode cycled in 1.0M‐DMFN (Figure S23f, Supporting Information) highlighting the poor stability and prominent loss of active material when cycled in E‐baseline. In addition, the 3‐D reconstruction of the cycled S electrodes corroborates the more highly agglomerated structure of the S electrode cycled in E‐baseline, revealing the collapse of the porous structure observed in the pristine electrode, while the DMFN cycled electrode retains smaller S particles and a porous electrode structure, as shown in Figure S24 (Supporting Information), respectively.

**Figure 6:**
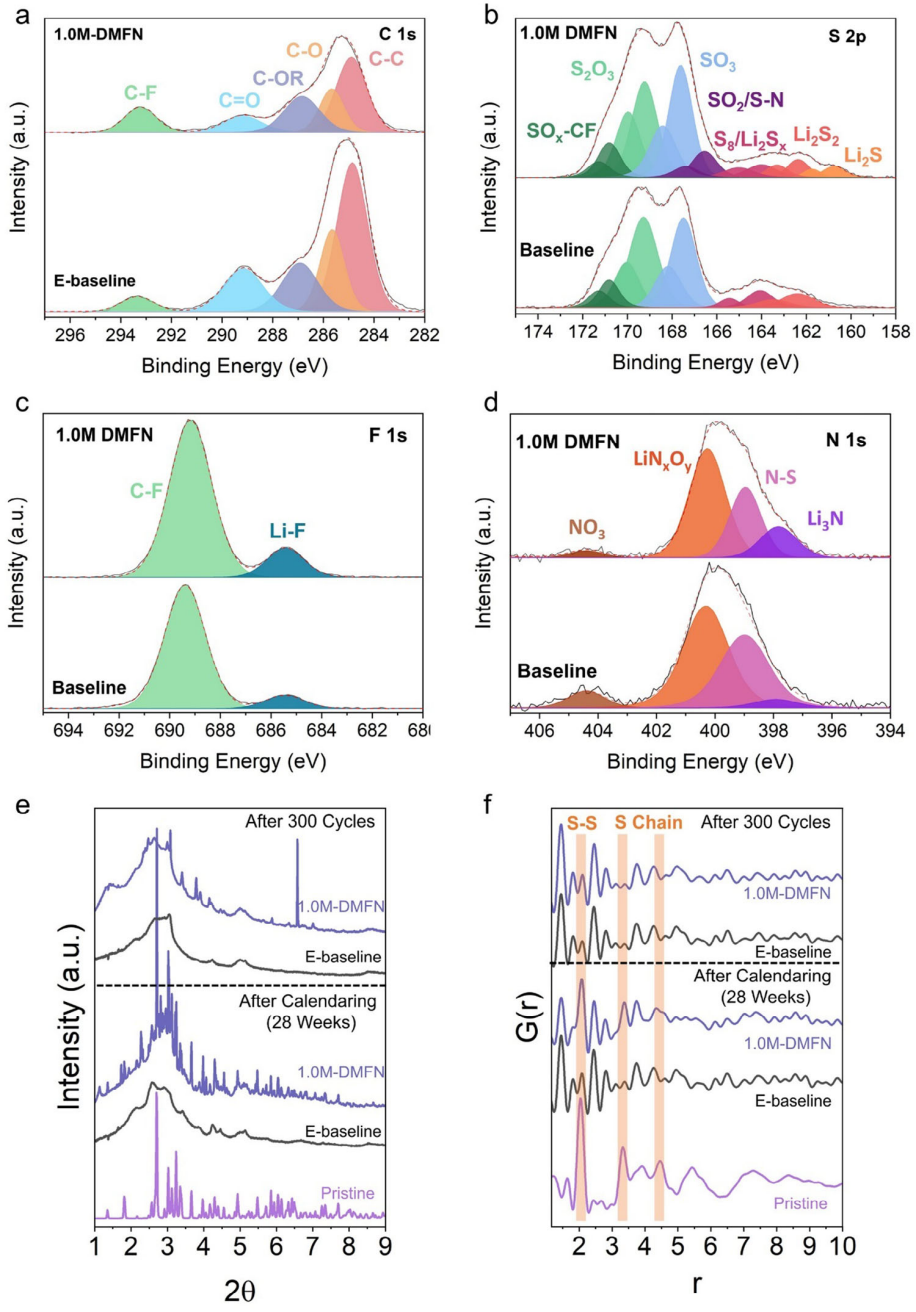
Post‐mortem characterization of S electrodes of pristine and 1.0M‐DMFN after 300 cycles at C/5. a–d) XPS analysis of C 1s, S 2p, F 1s, and N 1s spectra for cycled S electrodes from E‐baseline and 1.0M‐DMFN cells. e) XRD pattern and f) PDF of pristine S electrode and S electrodes after 300 cycles and 28 weeks calendaring in E‐baseline and 1.0M‐DMFN, respectively.

The XPS analysis of the DMFN cycled S electrode reveals a less prominent organic phase found in the C 1s (**Figure** **6**a), a higher intensity of peaks decomposed TFSI^−^ in the S 2p (Figure 6b), a slightly higher intensity of C–F and Li–F peaks in the F 1s spectra (Figure 6c) and a less prominent reduction of LiNO_3_ in the N 1s spectra (Figure 6d), indicating that the DMFN electrolyte promotes a more inorganic‐rich cathode electrolyte interphase (CEI), unlike the more organic‐rich and unstable CEI of the E‐baseline cycled electrode. The noticeably stronger C–C peak and the overall lower S intensity in the corresponding C 1s and S 2p spectra of the E‐baseline cycled electrode may be attributed to the bare C substrate and S particle delamination observed in the SEM images of the cycled electrodes, shown in Figure S22b (Supporting Information), corroborating the accelerated degree of LiPS dissolution and migration in E‐baseline. The improved morphology, structural integrity, and CEI composition observed of the cycled S electrode in 1.0M‐DMFN highlights the profound effect of the designed MSEs to reduce excessive LiPS dissolution and mitigate PS shuttling while cycling the LSB.

X‐ray diffraction (XRD) was performed on S electrodes in the pristine condition and after 300 cycles at C/5 rate and 28‐week period of calendaring and after one complete formation cycle in E‐baseline and 1.0M‐DMFN (Figure 6e; Figure S25 (Supporting Information), respectively). The XRD pattern after 300 cycles and calendaring period reveals the vastly improved retention of the S particles in the electrode when cycled or calendared in the 1.0M‐DMFN electrolyte, with significant degradation observed when combined in E‐baseline (Figure 6e). After one complete formation cycle, the DMFN cycled electrode has a mixture of α‐ and β‐S_8_, with overall stronger peak signal integrity, which is more comparable with the pristine S electrode. On the other hand, the E‐baseline cycled electrode has significant degradation in peak intensity with only peaks for β‐S_8_ was observed. The ring‐opening mechanism of the conversion of α‐ to β‐S_8_ is a well‐known phenomenon when converting S_8_ to Li_2_S_8_ and smaller LiPSs during discharge.^[^ ^6^, ^7^, ^18^ ^]^ Pair distribution function (PDF) analysis of the cycled and calendared electrodes (Figure 6f), corroborated the findings from XRD, showing enhanced peak retention and integrity of the S─S bond and S‐chains in the cycled and calendared electrodes in 1.0M‐DMFN, compared to those in E‐baseline. The designed DMFN electrolytes have significantly improved compatibility, reversibility, and retention of S particles and overall S electrode architecture with the observed morphology and microstructure measured after long‐term cycling and calendaring conditions. Further investigation utilizing in situ measurements^[^ ^49^, ^50^ ^]^ would be required to ascertain the mechanism of retaining α‐S_8_ in the DMFN after cycling, however, this observation could be a factor demonstrating an enhanced reversibility of S_8_ during cycling or improving long‐term stability and structural retention of the S‐electrode in the designed DMFN MSEs.

## 3 Conclusion

Inspired by the design of LHCEs for LMBs with conventional intercalation cathodes, a set of MSEs for LSBs has been designed by utilizing a ternary solvent system with a highly solvating solvent, a weakly solvating solvent, and a non‐coordinating diluent. These electrolytes limit the degree of LiPS solubility and in turn significantly enhance the long‐term cyclability and the calendaring performance of LSBs. The optimized MSE (1.0M‐DMFN) developed in this work exhibits superior Li compatibility and the highest average Li CE of 99.66%. LSBs with 1.0M‐DMFN can retain 81.3% capacity after 300 cycles at a C/5 charge/discharge rate, doubled the cycle life from the baseline electrolyte. The S and Li electrodes cycled in 1.0M‐DMFN can still maintain the structural integration and good morphology after 300 cycles and have more robust, anion‐ and F‐rich CEI and SEI layers, while the electrodes cycled in E‐baseline show serious corrosion and more organic species in their CEI and SEI. The DMFN electrolytes also demonstrate excellent stability during the calendar aging tests, with only 5.9% capacity loss over 28 one‐week resting intervals, while the baseline electrolyte has serious capacity loss in the initial cycles and another 23.5% loss during the 28‐week's calendar test. This indicates that the DMFN MSEs can significantly suppress the LiPSs shuttling, one of the most significant barriers in LSBs with conventional liquid electrolytes. This work sheds light for rationally design of a unique MSE solvation structure to enhance its stability with Li metal, mitigate the PS shuttle effect, and improve long‐term cycling and calendar stabilities of LSBs for their large‐scale, practical applications.

## Conflict of Interest

The authors declare no conflict of interest.

## Author Contributions

W.X. and D.J.K. conceived the idea for the project. D.J.K. and X.C. prepared the electrolytes and performed electrochemical experiments. P.G. conducted simulations and calculations. S.F. and D.L. prepared the S/C composite electrodes for coin cell tests. Q.Z. conducted XPS measurement. Y.X. J.P.Q. and C.W. performed the PFIB‐SEM and 3‐D reconstruction images. X.Z. did UV‐Vis measurements. M.M.R., S.T., and E.H. conducted XRD measurements. S.P., S.W., and Y.S.M. quantified the S and Li^0^ inventory. S.K. and D.F. assembled and tested the pouch cells. A.N. and J.W. prepared Li_2_S_6_ saturated solutions and did the elemental analysis, respectively. J.L. and J.‐G.Z. gave valuable suggestions on this work. All authors discussed the results and analyzed the data. D.J.K. and W.X. drafted the manuscript with revisions and comments from all authors.

## Supporting information



Supporting Information

## Data Availability

The data that support the findings of this study are available from the corresponding author upon reasonable request.
