# Supplementary material for: Designing Moderately‐Solvating Electrolytes for High‐Performance Lithium–Sulfur Batteries
Source: Adv Mater. 2025 Jun 4;37(33):2503365. doi: 10.1002/adma.202503365 (PMC12369677; doi:10.1002/adma.202503365)
Supplement: Supplementary file 1 — Supporting Information [file ADMA-37-2503365-s001.docx]

Supporting Information

**Designing Moderately-Solvating Electrolytes for High-Performance Lithium-Sulfur Batteries**

*David J. Kautz, Xia Cao, Peiyuan Gao, Shuo Feng, Qian Zhao, Saurabh Parab, Yaobin Xu, Joseph P. Quinn, Muhammad Mominur Rahman, Sha Tan, Xin Zhang, Sanaz Ketabi, Aqsa Nazir, Junxia Wang, Fang Dai, Shen Wang, Dongping Lu, Enyuan Hu, Y. Shirley Meng, Chongmin Wang, Jun Liu, Ji-Guang Zhang, and Wu Xu**

Dr. D. J. Kautz, Dr. X. Cao, Dr. S. Feng, Dr. Y. Xu, Dr. J. P. Quinn, Dr. A. Nazir, Dr. J. Wang, Dr. D. Lu, Dr. J. Liu, Dr. J.-G. Zhang, Dr. W. Xu

Energy and Environment Directorate, Pacific Northwest National Laboratory, Richland, WA 99354, United States

* E-mail: wu.xu@pnnl.gov

Dr. P. Gao, Dr. X. Zhang

Physical and Computational Sciences Directorate, Pacific Northwest National Laboratory, Richland, WA 99354, United States

Dr. Q. Zhao, Dr. C. Wang

Environmental Molecular Sciences Laboratory, Pacific Northwest National Laboratory, Richland, Washington 99354, United States

S. Parab, Dr. S. Wang, Dr. Y. S. Meng

Department of NanoEngineering, University of California San Diego, La Jolla, California 92093, United States

Dr. M. M. Rahman, Dr. S. Tan, Dr. E. Hu

Chemistry Division, Brookhaven National Laboratory, Upton, New York 11973, United States

Dr. S. Ketabi, Dr. F. Dai

General Motors Research and Development Center, Warren, Michigan 48092, United States

Dr. J. Liu

Materials Science and Engineering Department, University of Washington, Seattle, Washington 98195, United States

**Keywords:** lithium-sulfur battery, moderately solvating electrolyte, polysulfide, self-discharge, cycle and calendar life

**Materials and Methods**

*Electrolyte preparation*

The electrolytes were prepared by dissolving LiTFSI (battery grade, Gotion, Fremont, USA) and LiNO_3_ (battery grade, Sigma Aldrich, St. Louis, USA) in the selected solvent or solvent mixture [DME and DOL (battery grade, Gotion, Fremont, USA), 2-MeTHF (Sigma-Aldrich, St. Louis, USA) and TFEO (SynQuest Laboratories, Alachua, USA)] inside an MBraun glovebox filled with purified argon (Ar) and having moisture and oxygen content respectively less than 1 ppm. The DME and DOL were used as received, while LiTFSI was vacuum dried at 110 °C for overnight and the 2-MeTHF and TFEO were dried with pre-activated molecular sieves for at least one week before use and moisture content were measured with a Metrohm KF 851 titrator to ensure moisture content was below 10 ppm.

*Electrode preparation for coin cell testing*

The integrated Ketjen Black/sulfur (IKB/S) composite was prepared through a modified synthesis approach.^[1]^ Typically, KB powder (AkzoNobel) and poly(melamine-co-formaldehyde) methylated solution (Sigma-Aldrich) were thoroughly blended with a weight ratio of 1:1 and then the mixture was dried and carbonized at 900 °C under Ar atmosphere for 10 h. The collected IKB was heat treated with sulfur (weight ratio IKB:S=1:4) at 155 °C for 12 h, resulting in IKB/S.

For the electrode fabrication, carbon nanofiber (Sigma-Aldrich) was firstly dispersed in polyacrylic acid (Sigma-Aldrich) and dimethylformamide (DMF, Sigma-Aldrich) solution (2 wt.%) to form a uniform slurry. Then, the IKB/S particles were added and thoroughly mixed in a Thinky mixer for 15 minutes (ARE-310, Thinky). The weight ratio of IKB/S, carbon nanofiber and polyacrylic acid was controlled as 8:1:1 and the solid content in the slurry was 20%. The obtained slurry was casted onto aluminum foil with a S loading of 4 mg cm^-2^ and dried in a vacuum oven (half vacuum) at 60 °C for 12 h. 250-µm thick Li foil received from MSE supplies (Tucson, USA) was used as received.

*Electrode preparation for pouch cell testing*

Carbon/sulfur (C/S) composite was prepared by thoroughly blending Ketjen Black (KB EC600JD, Nouryon) and sulfur (Sigma-Aldrich). KB was milled and dried prior to sulfur addition (weight ratio KB:S=1:4). The mixture was heat treated at 155 °C for 10 h, resulting in C/S.

For the electrode fabrication, C/S composite, multi-walled carbon nanotubes (Gelon), and lithiated polyacrylic acid (Sigma-Aldrich) solution (6 wt.% in water) was mixed to form a uniform slurry. The weight ratio of C/S, multi-wall carbon nanotubes and lithiated polyacrylic acid was controlled as 9:0.5:0.5 and the solid content in the slurry was 28%. The obtained slurry was casted onto aluminum foil with a S loading of 4 mg cm^-2^ using roll to roll coating machine. Electrodes were dried in a vacuum oven at 60 °C for 12 h.

*Cell assembly and tests*

CR2032 coin cells were assembled for Li CE test and Li||S battery tests. The average Li CE was measured in the setup of Li||Cu configuration, with Li as anode and Cu as cathode, using the CE protocol (method 3 with *Q_T_* of 5 mAh cm^−2^, *Q_C_* of 1 mAh cm^−2^ and *n* of 10) reported in our previous work.^[2]^ Li||S cells were assembled for the cycling and self-discharge tests. For all the cells, polyethylene separator (Asahi Hi-Pore, Japan) and electrolyte of 75 µL for Li||Cu cells or electrolyte/sulfur (E/S) ratio of 8 µL mg^-1^-S for Li||S cells were applied. The Li||S cell cycling stability was tested within a voltage range of 1.7–2.8 V at C/10 or C/5 after 2 formation cycles at C/20 in the voltage range of 1.8-2.8 V. For the self-discharge of the Li||S cells, the cells were first performed for 2 formation cycles at C/20 in 1.8-2.8 V, then charged to 2.8 V at C/10 and followed with a 168-h rest, and after that discharged to 1.7 V at C/10 and repeated for 28 intervals. All cells were cycled on Landt battery testers at 25 °C in TestEquity TEC1 thermoelectric environmental chamber.

1 Ah pouch cells were fabricated with 100-µm thick Li foil received from Albemarle. Pouch cells were filled with E-baseline and 0.8M-DMFN electrolytes at E/S of 5.2, and then assembled into cell fixture with pressure set at 25 PSi (fixed-gap cell fixture configuration). The cells were first cycled at C/20 for 2 formation cycles in 1.8 – 2.6 V followed by C/10 rate in 1.7-2.6 V at 25 °C.

*Classical molecular dynamic (CMD) Simulation*

All the CMD simulations were carried out with the GROningen MAchine for Chemical Simulations (GROMACS) simulation package. The details of the simulation systems and interaction parameters are included in SI. Initially the molecules and ions were randomly inserted into the simulation box. The steepest descent method was used to minimize the energy of the systems. The systems were pre-equilibrated in isothermal–isobaric (NPT) ensemble with 10 ns at 298 K and 1 bar. The temperature and pressure were controlled by V-rescale thermostat^[3]^ and Berendsen barostat^[4]^ with a time constant of 0.2 and 1 ps. Then 200 ns production simulations were performed at 298 K in canonical (NVT) ensemble. The temperature and pressure were controlled by the Nose-Hoover themostat^[5]^ with a time constant of 0.2 ps. The cutoff of the Lennard-Jones potential was 1.2 nm. The particle mesh Ewald method^[6]^ with a Fourier spacing of 0.15 nm and a 1.2 nm real-space cutoff were used for calculating electrostatic interactions. Periodic boundary conditions were used in all three directions. The time step was 2 fs. The bonds between H and other atoms were constrained by the LINCS algorithm.^[7]^ The snapshots were generated by Visual Molecular Dynamics (VMD) software.^[8]^

*Density function theory (DFT) calculation*

The highest occupied molecular orbital (HOMO) and the lowest unoccupied molecular orbital (LUMO) energies and the local softness descriptors of solvent and diluent molecules are determined using DFT calculations. The approach for the calculations follows the methodology used in our previous work.^[9,10]^

*Characterizations*

Saturated Li_2_S_6_ solutions were prepared by combining stoichiometric amounts of Li_2_S and S_8_ (8:7) in the chosen solvent mixture, followed by stirring at 55 °C for 20 h within the glovebox. After this period, photographs were taken of the solution mixtures. Subsequently, the mixtures were centrifuged to isolate the clear liquid phase and then diluted 100-fold using the same solvent mixture used in the electrolyte. The diluted solutions were transferred into Globe Scientific Spectrophotometer Cuvettes (Fisher Scientific) and analyzed using a UV-Vis spectrophotometer (UV-Vis 2501PC, Shimadzu) at a scan rate of 0.5 nm s^-1^, covering a wavelength range from 800 to 200 nm.

Elemental analysis of saturated Li_2_S_6_ solutions was conducted using an Elementar Vario Macro Cube. This instrument automatically determines C-H-N-S by combustion of the sample, separation of the combustion products by means of a programmed temperature desorption system, and measurement by thermal conductivity. Combustion and reduction tubes were packed accordingly to analyze carbon, nitrogen, sulfur, and hydrogen. The combustion tube was heated to 1150 °C and the reduction tube to 850 °C. Helium was used as the carrier gas. Typical sample sizes ranged from 10–30 μL.

For postmortem analyses, including scanning electron microscopy (SEM) and X-ray photoelectron spectroscopy (XPS), the tested coin cells were disassembled inside the glovebox to receive the cycled Li anodes and S cathodes. These electrodes were subsequently rinsed with DME solvent to eliminate the residual electrolytes, vacuum dried, and sealed in airtight containers within the glovebox before being transferred for further characterizations. SEM measurements were conducted using a JEOL JSM-IT200 at an accelerating voltage of 5 kV and a current of 86 pA. XPS analysis was conducted on a Physical Electronics Quantera Scanning X-ray Microprobe (Physical Electronics, Germany), which used a focused monochromatic Al Kα (1486.7 eV) source for excitation. High-energy resolution S 2p and F 1s spectra were collected using a pass-energy of 69 eV with a step size of 0.125 eV. The obtained spectra were fitted with the CasaXPS software with the binding energy was calibrated with C 1s at 284.8 eV. 3D slicing using cryogenic (cryo) plasma focused ion beam (PFIB)-SEM was carried out to reconstruct the 3D S structure on Helios 5 Hydra DualBeam (ThermoFisher Scientific). The S electrode was collected in the Ar glovebox and then transferred to the cryo PFIB-SEM chamber with inert gas transfer holder to avoid air contamination. After that, the SEM stage was cooled to -190 °C and the 3D-slice images/energy dispersive spectroscopy maps were acquired at cryogenic temperature. This process was performed using ThermoFisher Auto Slice & View 4 software. SEM image stacks were aligned and segmented using MATLAB’s Image Processing Toolbox. The alignment was performed using image registration algorithms with a one-plus-one evolutionary optimizer configuration and a mean squares error metric configuration. Segmentation was performed using gray thresholding and adaptive gray to remove shadowing effects. The gray thresholds were manually selected to differentiate material and void space. MATLAB was also used for 3D volume visualization. For X-ray diffraction (XRD) and pair distribution function (PDF) measurements, sulfur powder was harvested after Li-S battery cycling and packed inside the polyimide capillary (Cole-Parmer) with both sides sealed by epoxy glue inside Ar-filled glovebox. The XRD and PDF experiments were performed at the 28-ID-2 beamline of the National Synchrotron Light Source II of Brookhaven National Laboratory, using a photon wavelength of 0.1819 Å. The raw data were integrated using Dioptas software^[11]^, followed by intensity correction, background removal, and Fourier transform in PDFgetx3 to obtain the PDF data.^[12]^

**Supplementary properties of studied electrolytes**


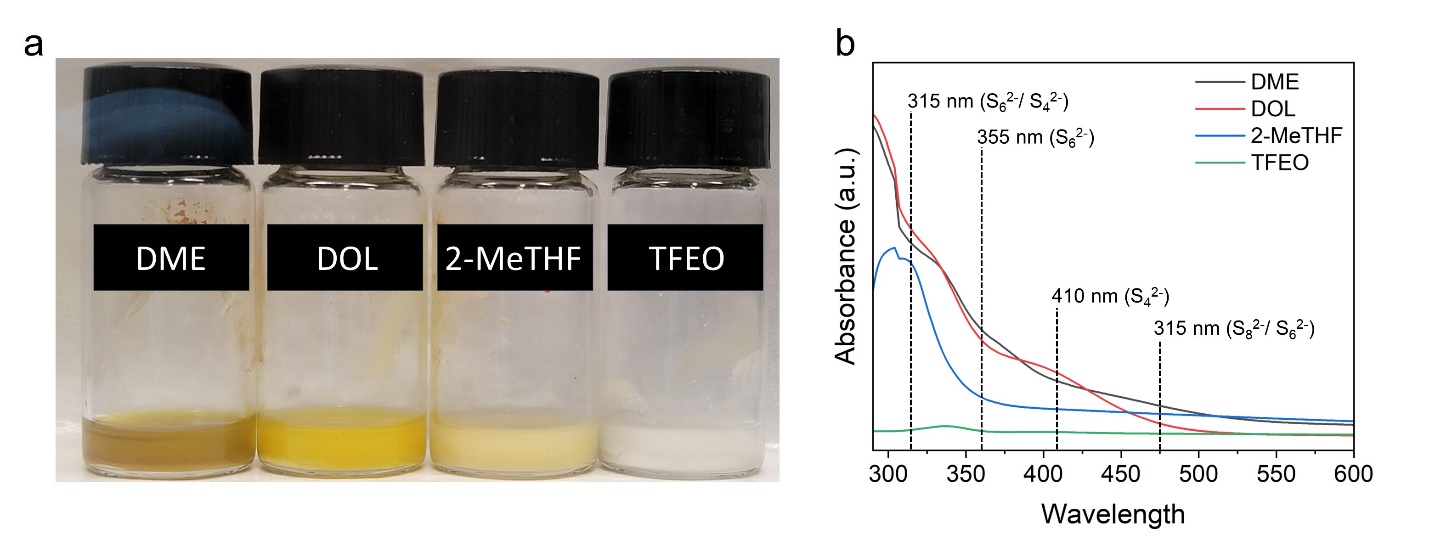


**Figure S1.** Photograph and UV-Vis absorbance of 0.1 M Li_2_S_6_ in selected solvents. (a) Photograph of 0.1 M Li_2_S_6_ in DME, DOL, 2-MeTHF, and TFEO after stirring at 55 °C for 20 h inside an Ar-filled glovebox. (b) UV-Vis spectra of 0.1 M Li_2_S_6_ in different electrolytes after stirring at 55 °C for 20 h.


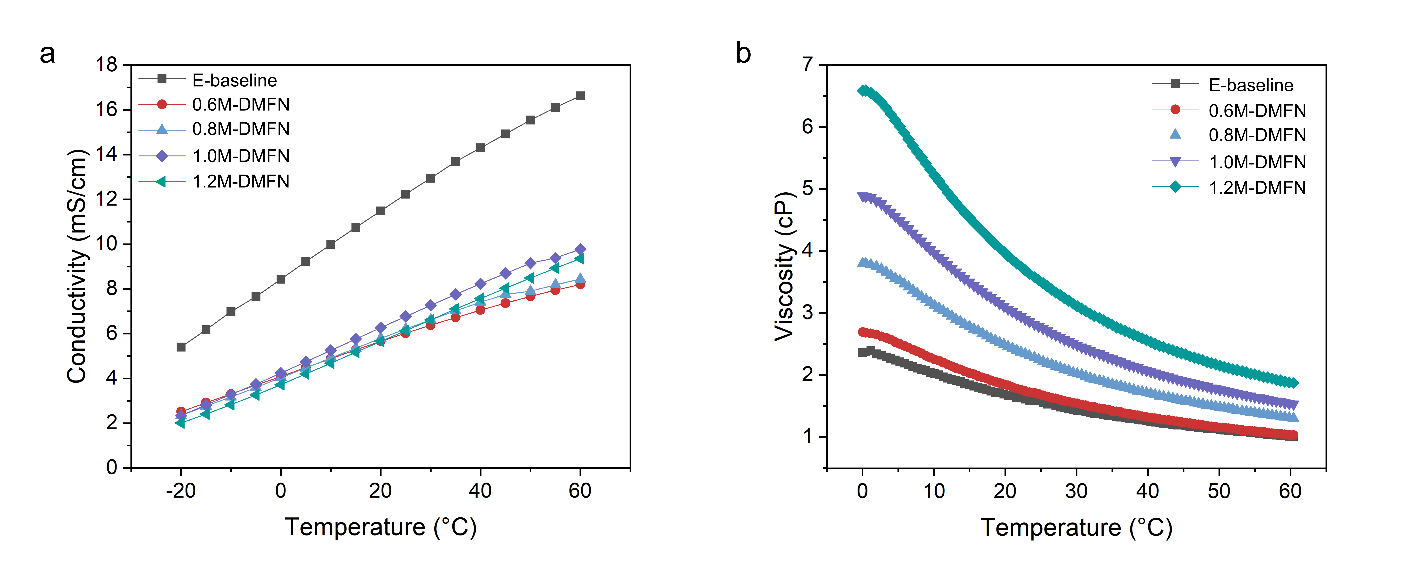


**Figure S2.** (a) Temperature dependence of ionic conductivity of E-baseline and DMFN electrolytes in a temperature range of -20 – 60 °C with measurement at every 5 °C. (b) Viscosity variation of E-baseline and DMFN electrolytes in a temperature range of 0 – 60 °C.


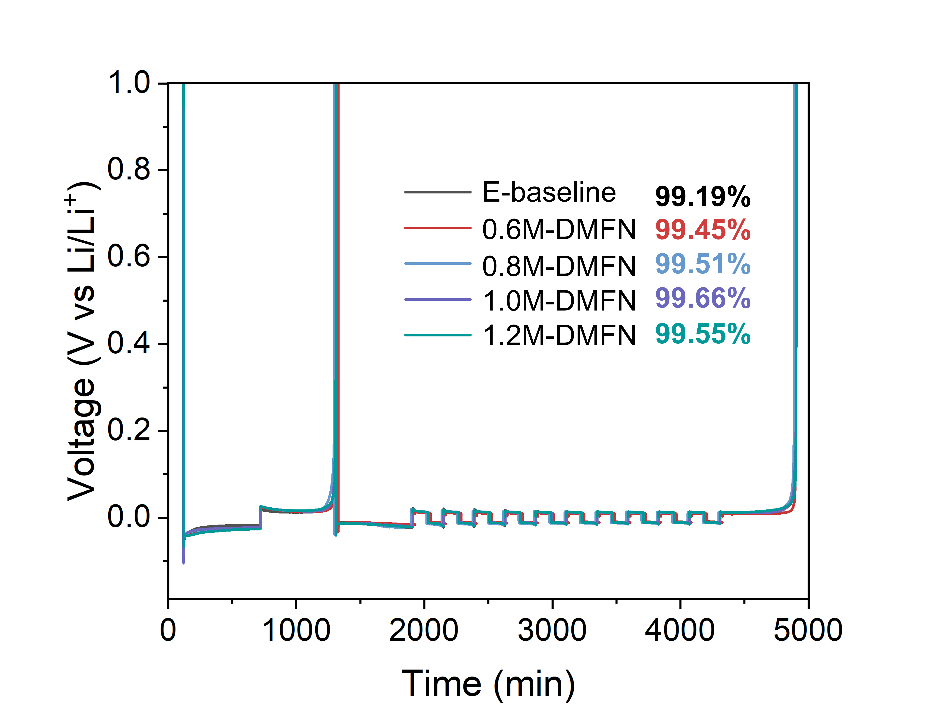


**Figure S3.** Voltage profiles of Li||Cu cells with E-baseline and DMFN electrolytes for measuring the average Li CE values.


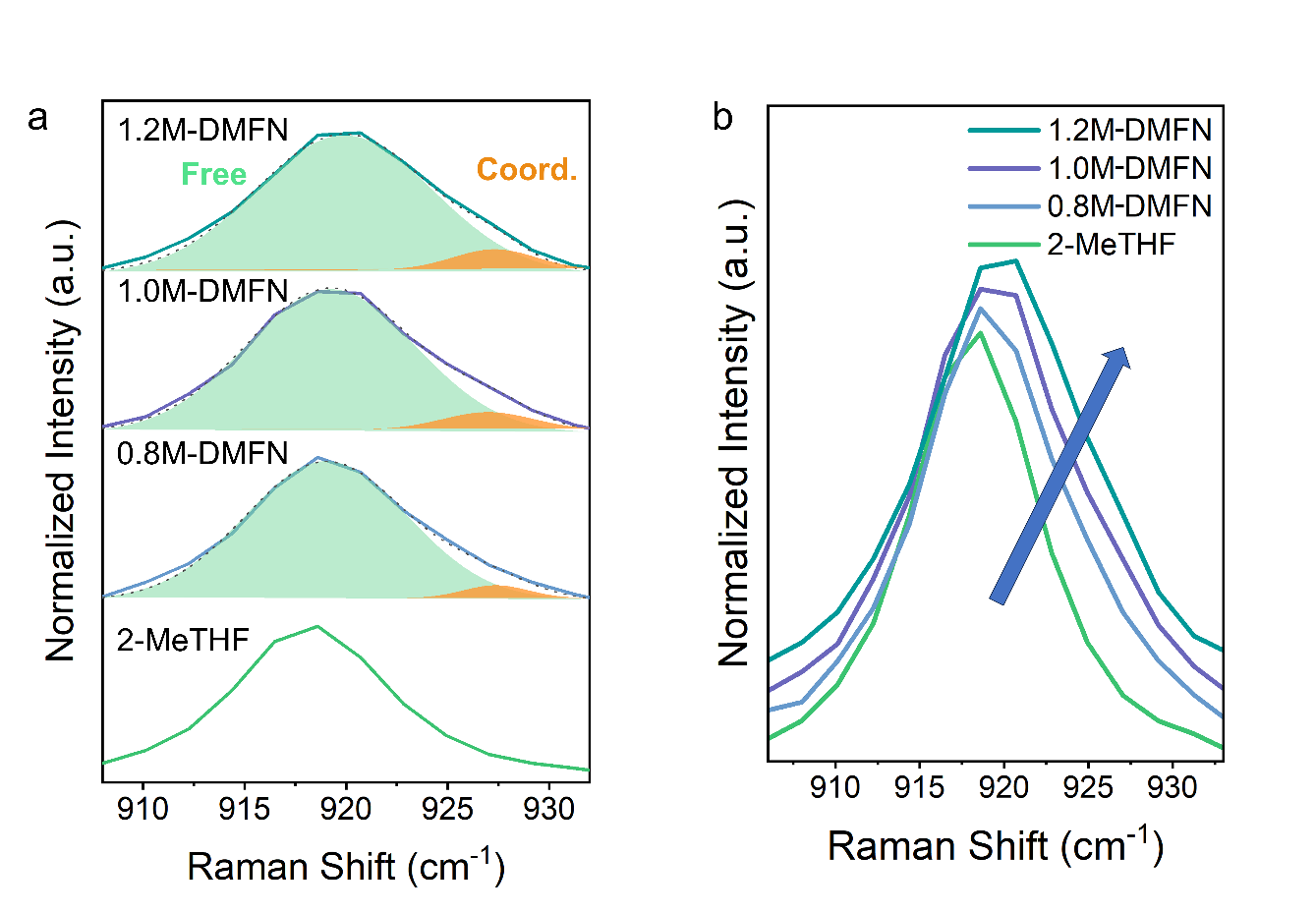


**Figure S4.** Raman spectra of pure 2-MeTHF and DMFN electrolytes comparing (a) free and coordinated 2-MeTHF and (b) growth of shoulder associated with coordinated 2-MeTHF with increasing LiTFSI concentration.


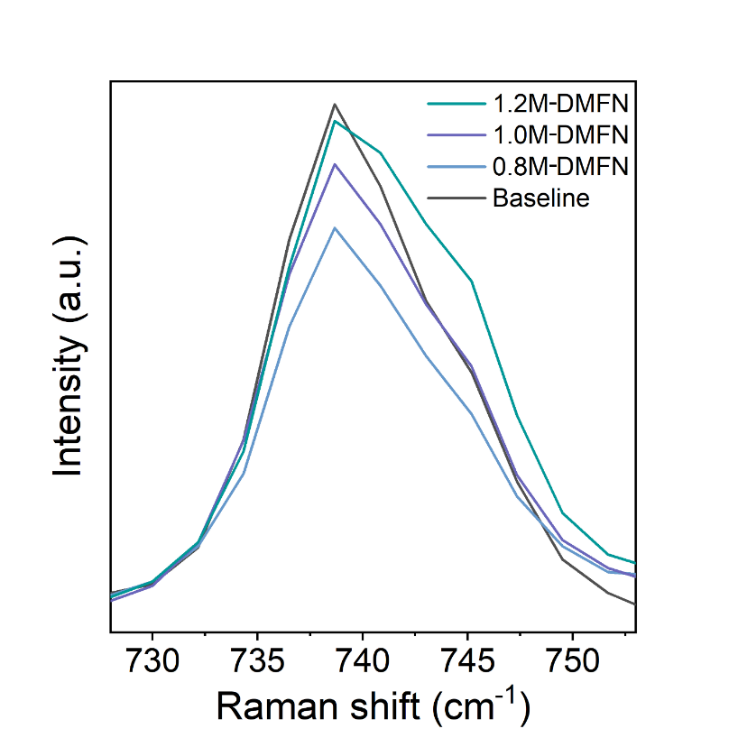


**Figure S5.** Raman spectra of stacked LiTFSI peak for E-baseline and DMFN electrolytes.

**Simulation systems and interaction parameters in classical molecular dynamics simulation**

We followed the combination approach of Dzubiella for the interaction parameters. ^[13]^ In Dzubiella’s combination approach, the parameters of TFSI^-^ and NO_3_^-^ anion were obtained from CL&P force field.^[14]^ The parameters of Li ion were obtained from Dang’s work.^[13]^ The parameters of polysulfide were from Persson’s work.^[15,16]^ The interaction parameters for the four solvents (DME, DOL, 2MeTHF and TFEO) in our simulation were obtained from optimized potentials for liquid simulations (OPLS) force field.^[17,18]^ This combination of force field parameters has been validated to reproduce very well experimental diffusion coefficients, conductivity, viscosity, and density of similar solutions.^[19,20]^ In this work, the F and hydrogen (H) interaction parameters were tuned^[21]^ to improve the excess volume and interfacial enthalpies of fluorinated ether. The Li-S interaction parameter has been optimized to reproduce the structure in neutron scattering experiment.^[22]^

To include the electronic polarizability effect, the partial charges of ions were treated by electronic continuum correction (ECC) method,^[23]^

$q_{i}^{eff}=\frac{q_{i}}{\sqrt{\varepsilon_{\infty}}}$ (1)

Here, $\varepsilon_{\infty}$ is the high-frequency contribution to the solvent permittivity stemming from electronic fluctuations in the solvent molecules. It is be related to the refractive index *n* as

$\varepsilon_{\infty}=n^{2}$ (2)

The refractive indices of solvents are listed in Supplementary Table S1. The refractive indices of mixtures were calculated by Arago-Biot approach.^[24]^

**Table S1.** The refractive indices of solvents

| Solvent | Refractive index |
| --- | --- |
| 2MeTHF ^[25]^ | 1.407 |
| DME ^[26]^ | 1.378 |
| DOL ^[27]^ | 1.398 |
| TFEO ^[28]^ | 1.3203 |

The compositions of the simulation systems are based on the experimental setup. To investigate the solubility of Li polysulfide in electrolytes, Li_2_S_6_ was selected as the example. Li_2_S_6_ molecules (60-180) were added to each electrolyte systems to simulate the electrolytes with various Li_2_S_6_ concentrations.

**Supplementary simulations on coordination environment of studied electrolytes**


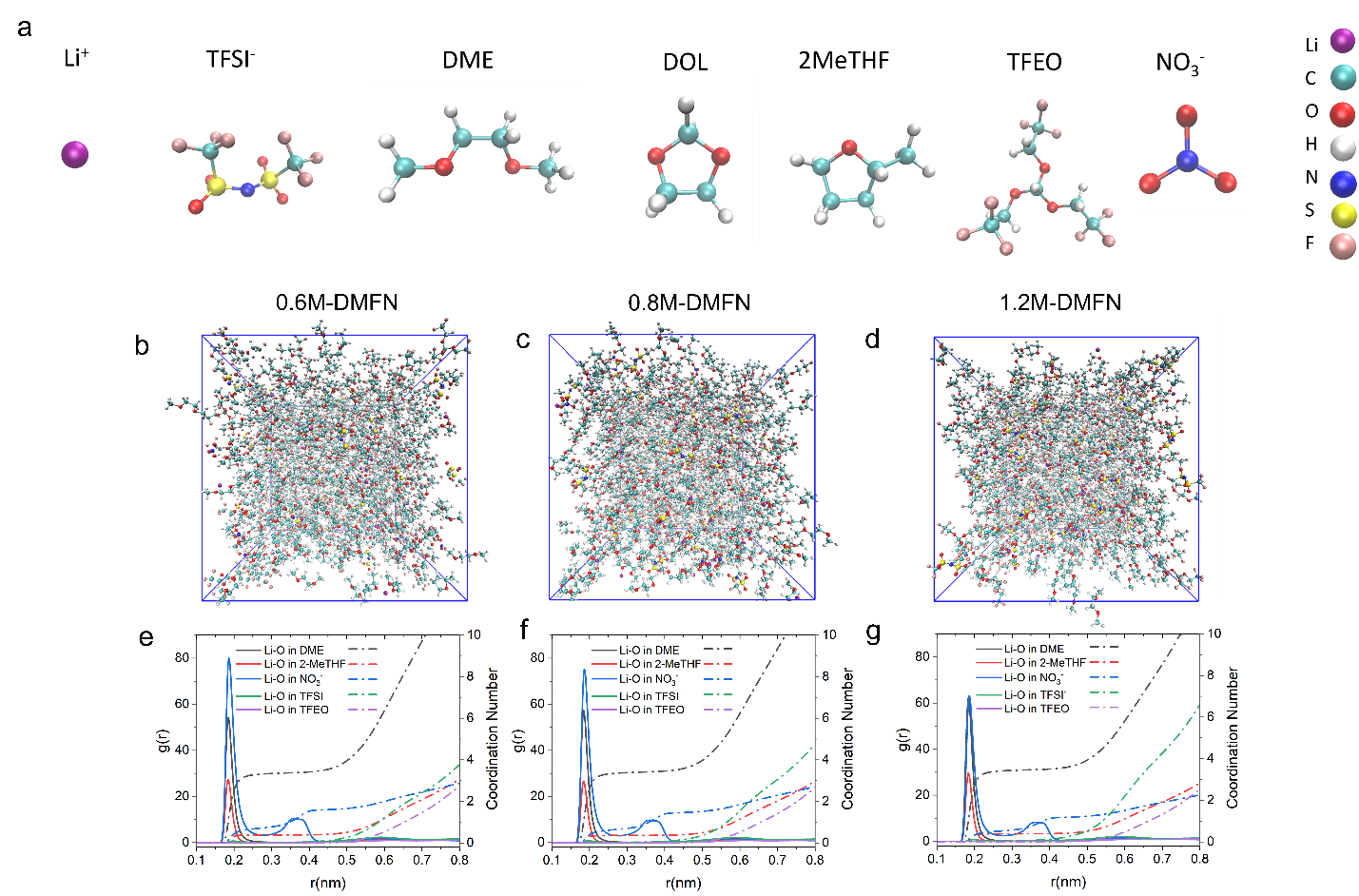


**Figure S6.** Molecular dynamics solvation investigation of studied electrolytes. (a) Molecular structures of Li^+^, anions, and solvents of E-baseline and DMFN electrolytes. (b-d) Classical molecular dynamics (CMD) simulation snapshots of 0.6M, 0.8M, and 1.2M DMFN electrolytes**.** (e-g) Radical distribution function curves and coordination numbers of Li-O bonds in solvents and anion pairs calculated from CMD simulation for the corresponding electrolytes.

In addition to the Li_2_S_6_ solubility test, a piece of the S electrode was placed into 1 mL of the E-baseline and 1.0M-DMFN electrolytes over a period of two months to observe the solubility and innate diffusion of S_8_ from the pristine S electrode into the electrolyte (Supplementary Fig. S6). After three days of soaking, a faint yellow color was observed in E-baseline which turned into darker yellow after one week and remained a similar dark yellow color for two months, indicating that the solution was fully saturated from the diffusion of the S from the electrode. For the 1.0M-DMFN electrolyte with S electrode,


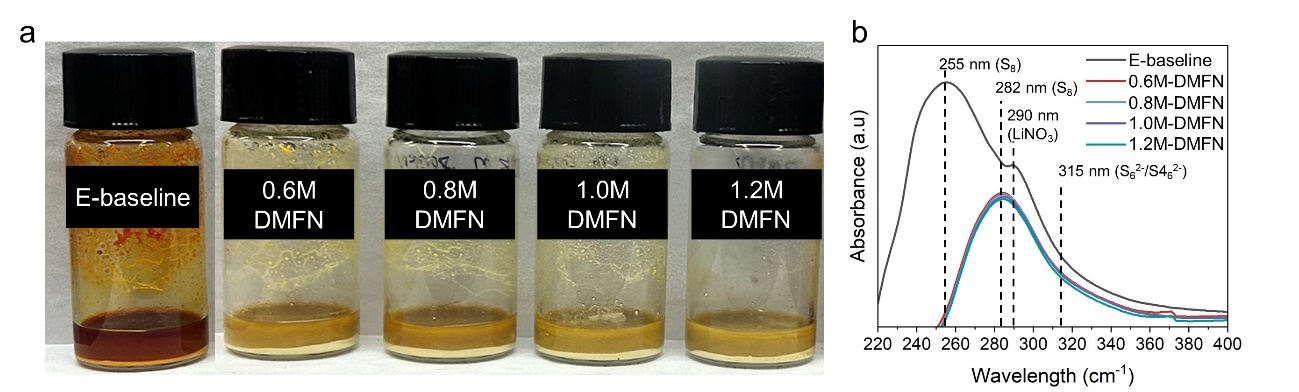


**Figure S7.** Photograph and UV-Vis absorbance of 1.5 M Li_2_S_6_ in studied electrolytes. (a) Photograph of 1.5 M Li_2_S_6_ in different electrolytes after stirring at 55 °C for 20 h inside an Ar-filled glovebox. (b) UV-Vis spectra of 1.5 M Li_2_S_6_ in different electrolytes after stirring at 55 °C for 20 h.

**Table S2.** Average weight percentages of elements in electrolyte samples with saturated Li_2_S_6_

| Sample code | Average weight percentage of elements in electrolyte samples | | | |
| --- | --- | --- | --- | --- |
|  | C (%) | H (%) | N (%) | S (%) |
| E-baseline | 32.315 ± 0.045 | 4.919 ± 0.202 | 1.700 ± 0.050 | 15.472 ± 0.320 |
| 0.6M-DMFN | 31.200 ± 0.750 | 4.276 ± 0.123 | 1.965 ± 0.105 | 12.499 ± 0.712 |
| 0.8M-DMFN | 30.938 (+2.493, −2.788) | 4.471 (+0.465, −0.649) | 1.770 (+0.510, −0.200) | 10.012 (+0.652, −1.882) |
| 1.0M-DMFN | 32.030 ± 0.340 | 4.566 ± 0.066 | 1.890 ± 0.140 | 12.281 ± 0.554 |
| 1.2M-DMFN | 31.440 ± 0.050 | 4.319 ± 0.050 | 1.880 ± 0.040 | 13.486 ± 0.029 |

It is seen from Table S2 that the total S content in E-baseline is higher than those in the four DMFN electrolytes, meaning more Li_2_S_6_ exists in E-baseline. Interestingly, with the increase of LiTFSI concentration from 0.6 M to 1.2 M in the electrolyte, the Li_2_S_6_ content decreases first and then increases and shows the lowest Li_2_S_6_ solubility at 0.8 M, though the reason is unknown now. However, there are two sources of S and N in the electrolyte samples, like LiN(**S**O_2_CF_3_)_2_ and Li_2_**S**_6_ for **S** while Li**N**(SO_2_CF_3_)_2_ and Li**N**O_3_ for **N**, respectively. Since the actual molar concentrations of LiTFSI, Li_2_S_6_ and LiNO_3_ in these Li_2_S_6_-saturated electrolytes are unknown, it is impossible to get the exact Li_2_S_6_ concentrations in these electrolytes even through the calculations from the N and S contents by CHNS combustion analysis.

In another effort, two saturated solutions of Li_2_S_6_ in DME-DOL (1:1 by vol) and DME-2MeTHF-TFEO (2:1:1 by vol) that are the solvent mixtures for E-baseline and DMFN electrolytes, respectively, were prepared for elemental analysis. The C-H-S contents in the two solutions (or the two solvent mixtures) are summarized in Table S3. It is seen that the S content in the solvent mixture for E-baseline is significantly higher than that in the solvent mixture for DMFN electrolytes.

**Table S3.** Average weight percentages of elements in solvent mixtures with saturated Li_2_S_6_

| Solvent sample | Average weight percentages of elements | | |
| --- | --- | --- | --- |
|  | C (%) | H (%) | S (%) |
| DME-DOL (1:1 by vol) | 39.080  (+0.930, −0.900) | 7.022  (+0.212, −0.214) | 17.240  (+0.310, −0.329) |
| DME-2MeTHF-TFEO (2:1:1 by vol) | 34.993  (+4.477, −5.163) | 5.436  (+0.739, −0.842) | 7.478  (+0.344, −0.303) |

To obtain the concentrations of Li_2_S_6_ in the two solvent mixtures, simple calculations were performed as shown in Table S4. It is seen that the maximum molar concentrations of Li_2_S_6_ in the two solvent mixtures are 0.83 M in DME-DOL (1:1 by vol) for E-baseline and 0.38 M in DME-2MeTHF-TFEO (2:1:1 by vol) for DMFN electrolytes. As is well known, when there exists a solute in the solution, like LiTFSI, the later added salt (i.e. Li_2_S_6_ in this study) will have lower concentration when compared to that in the solution without the first solute. Therefore, the Li_2_S_6_ maximum concentration in DMFN electrolytes will be even lower than 0.38 M, falling in the moderate concentration range. Of course, more appropriate analysis techniques should be used to quantify the Li_2_S_6_ concentration in real electrolytes already with LiTFSI salt and LiNO_3_ additive.

**Table S4.** Calculations of maximum concentrations of Li_2_S_6_ in solvent mixtures

| **Liquid sample** | | | **S** | | | **Li_2_S_6_** | | | |
| --- | --- | --- | --- | --- | --- | --- | --- | --- | --- |
| Solvent mixture | Volume (µL) | Weight (mg) | Wt.% | Weight (mg) | Mole (mmol) | Mole (mmol) | Weight (mg) | Wt.% | Conc. (M) |
| DME-DOL | 12 | 11.15 | 17.240 | 1.922 | 0.0599 | 0.0100 | 2.063 | 18.500 | 0.83 |
| DME-2MeTHF-TFEO | 12 | 11.87 | 7.478 | 0.888 | 0.0277 | 0.0046 | 0.949 | 7.994 | 0.38 |

**
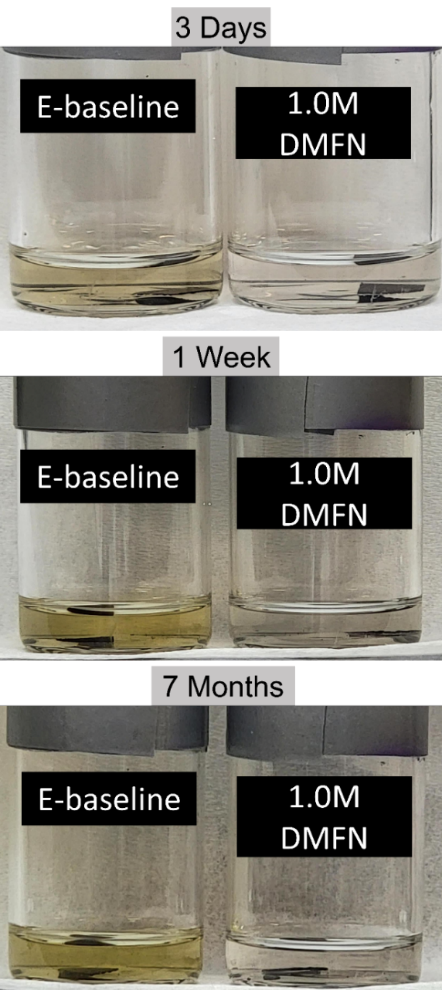
**

**Figure S8.** Photograph of S electrode soaked in the E-baseline and 1.0M-DMFN electrolytes after soaking for a 3-day, 1-week, and 7-month period.

The solvation environments of LiPSs were modeled by CMD simulations for Li_2_S_6_ in the electrolytes as the representative PS to further understand the interactions between the LiPSs with the different electrolytes. The PS cluster size, properties, and radial distribution function (RDF) in varying concentrations of Li­_2_S_6_ solutions were modeled in E-baseline and all four DMFN electrolytes, as shown in Figs. S9-S13. The first modeled environment consisted of 60 molecules of Li_2_S_6_ in the solution and it is observed that the distribution of Li_2_S_6_ clusters and average number of S atoms in each cluster is consistent for E-baseline and in all DMFN electrolytes with varied concentrations (Fig. S9a). As the concentration of the Li_2_S_6_ is increased to 120 and 180 molecules in the solution, the distinction between the E-baseline and the DMFN electrolytes becomes more apparent. The modeled probability of the Li_2_S_6_ cluster distribution in solution reveals that there is a greater probability of smaller clusters formation in E-baseline and larger Li_2_S_6_ clusters formed in the DMFN electrolytes signifying the lower solubility of LiPSs in the DMFN electrolytes (Fig. S9b,c).


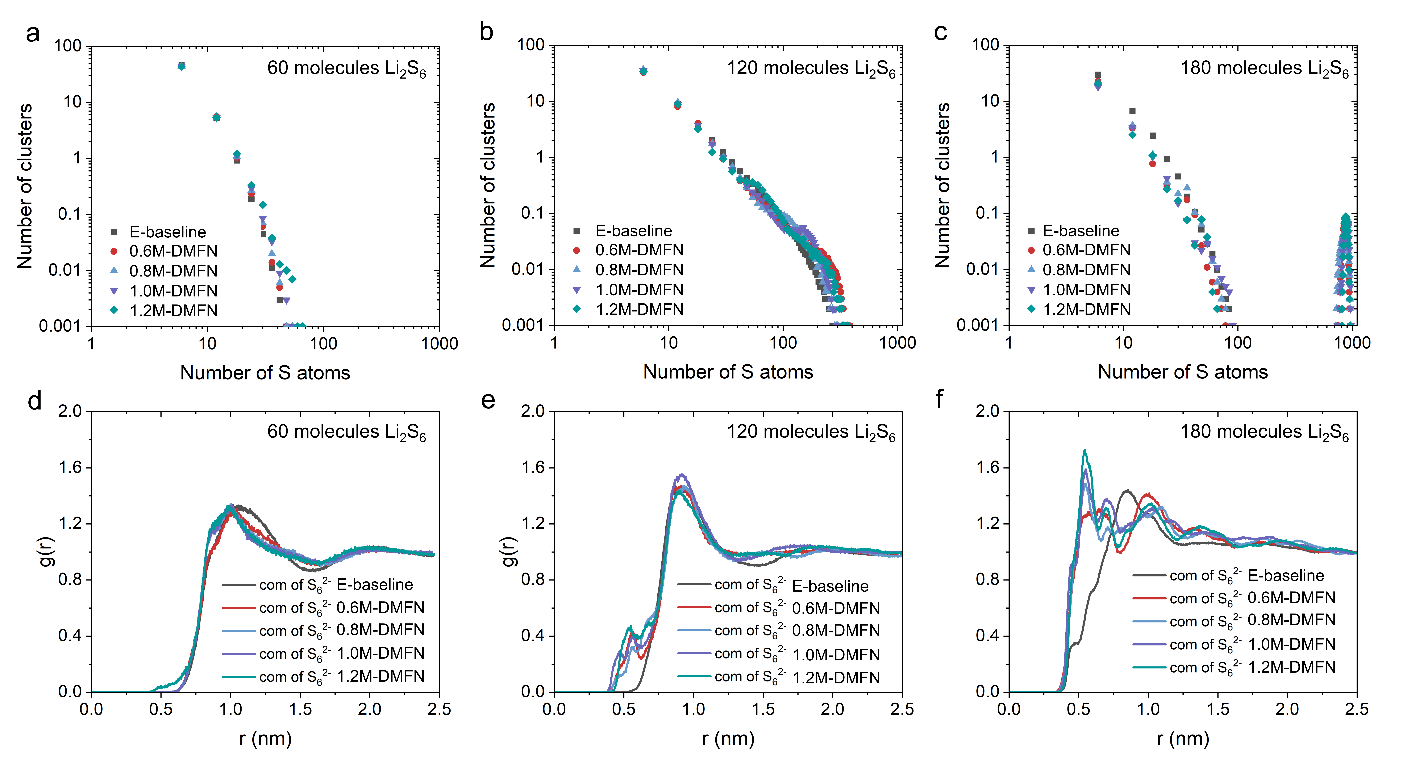


**Figure S9.** Polysulfide solubility in studied electrolytes. (a-c) Simulated cluster analysis of Li_2_S_6_ in electrolytes. (d-f) RDF of the center of mass (com) of S_6_^2-^ of Li_2_S_6_ in E-baseline and DMFN electrolytes.

The RDF of the center of mass of S_6_^2-^ also shows a clear distinction of the clustering properties between E-baseline and DMFN electrolytes. At Li_2_S_6_ = 60 there is no significant difference between all electrolytes as the LiPS is sufficiently solvated and well dispersed in the electrolytes (Fig. S9d). However, as the Li_2_S_6_ concentration was increased, a new peak was observed when Li_2_S_6_ = 120 at r = 0.5 nm for the DMFN electrolytes and the peak intensity increased substantially at Li_2_S_6_ = 180 (Fig. S9e,f). The peak at lower distance indicates the formation of more closely packed Li_2_S_6_ clusters due to the lower solubility in the DMFN electrolytes that become increasingly more densely packed at higher concentration, which is only observed in E-baseline at the highest Li_2_S_6_ = 180 with a weak intensity. The average number of atoms of the LiPS clusters reveals that in various electrolytes, there is no observed significant difference for the number of atoms in a cluster (Fig. S10), but the properties of the clusters overall highlight the lower solubility of Li_2_S_6_ in the DMFN electrolytes than in the conventional DME-DOL baseline electrolyte.


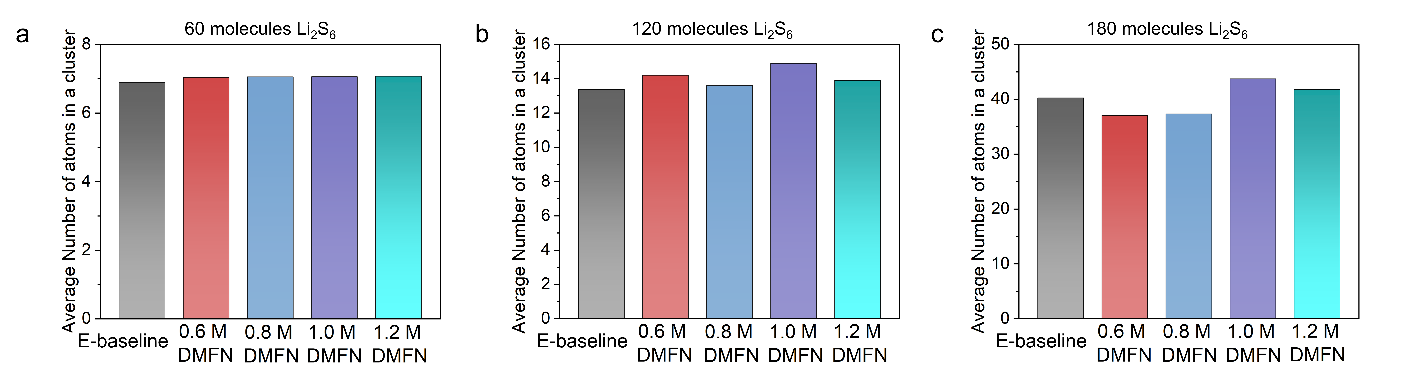


**Figure S10.** Simulated analysis of average number of atoms of Li_2_S_6_ clusters for (a) 60, (b) 120, and (c) 180 Li_2_S_6_ molecules in the corresponding electrolytes.

Simulated snapshots and RDF curves of the Li_2_S_6_ = 60 and 180 solutions were plotted to further delve into the coordination environments of the E-baseline and DMFN electrolytes (Figs. S11 and S12). The RDF results further corroborate that in the lower Li_2_S_6_ concentration solutions the Li_2_S_6_ molecules are effectively dissolved and dispersed in the electrolytes with the E-baseline and DMFN electrolytes having a low intensity of the Li-S bond in the first solvation shell and more abundant coordination presenting in the Li-O solvent bonds in all electrolytes. However, for the Li_2_S_6_ =180 solution, the DMFN electrolytes are shown to have a higher intensity of Li-S bonding in the first solvation shell for Li_2_S_6_. This result is related to the increased degree of clustering of the LiPS in the electrolyte, implying that the DMFN electrolytes are saturated with the LiPS, meanwhile the E-baseline is not fully saturated and has the capability for further dissolution of the LiPS. The RDFs for both the Li-S bond and the center of mass of S_6_^2-^ were calculated in pure TFEO revealing there is no solvation of or interaction between PSs and the TFEO solvent (Fig. S13). The design of the moderately solvating DMFN electrolytes, utilizing the weakly solvating solvent 2-MeTHF and non-coordinating solvent TFEO, reduces the overall solubility of and interactions with PSs, compared to the conventional DOL/DME baseline electrolyte that has an abundant capability for PS dissolution and interaction.


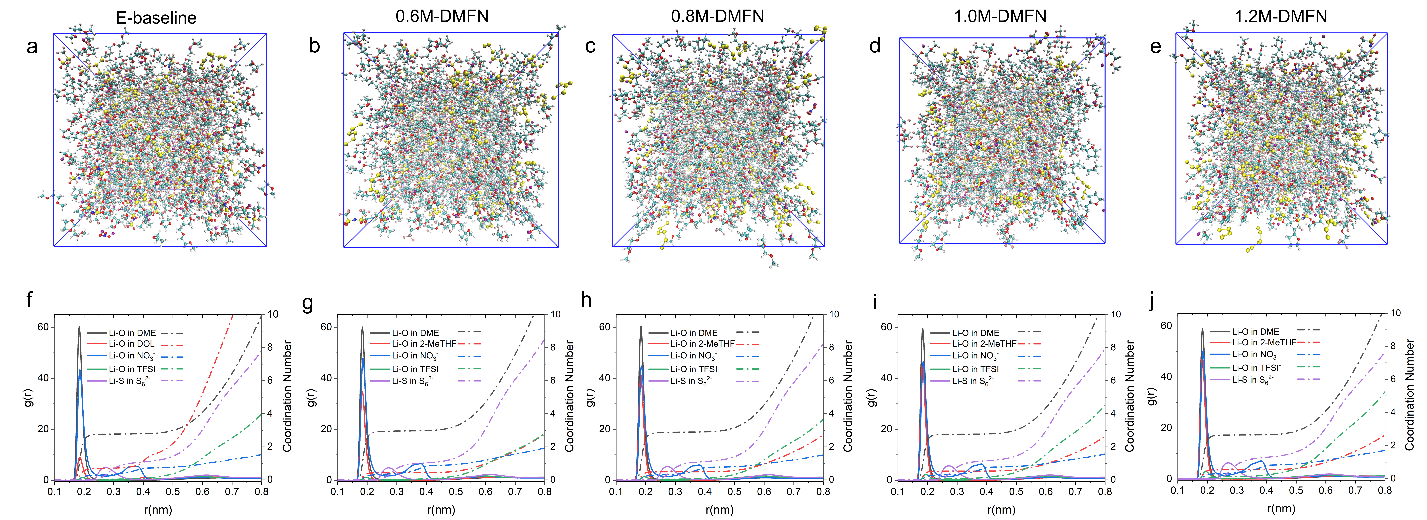


**Figure S11.** Molecular dynamics solvation investigation of studied electrolytes with 60 molecules of Li_2_S_6_ in solutions. (a-e) CMD simulation snapshots of E-baseline and DMFN electrolytes. (f-g) RDF and coordination numbers of Li-O bonds in solvents and anion pairs and Li-S bonds of Li_2_S_6_ calculated from CMD simulation for the corresponding electrolytes.


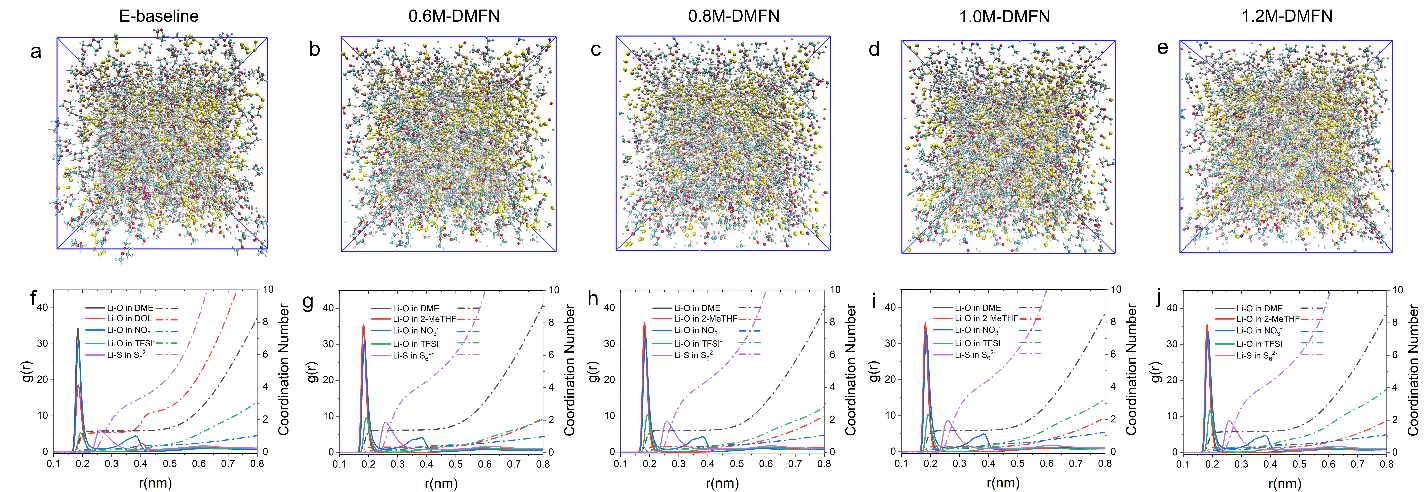


**Figure S12.** Molecular dynamics solvation investigation of studied electrolytes with 180 molecules of Li_2_S_6_ in solutions. (a-e) CMD simulation snapshots of baseline and DMFN electrolytes. (f-g) RDF and coordination numbers of Li-O bonds in solvent and anion pairs and Li-S bonds of Li_2_S_6_ calculated from CMD simulation for the corresponding electrolytes.


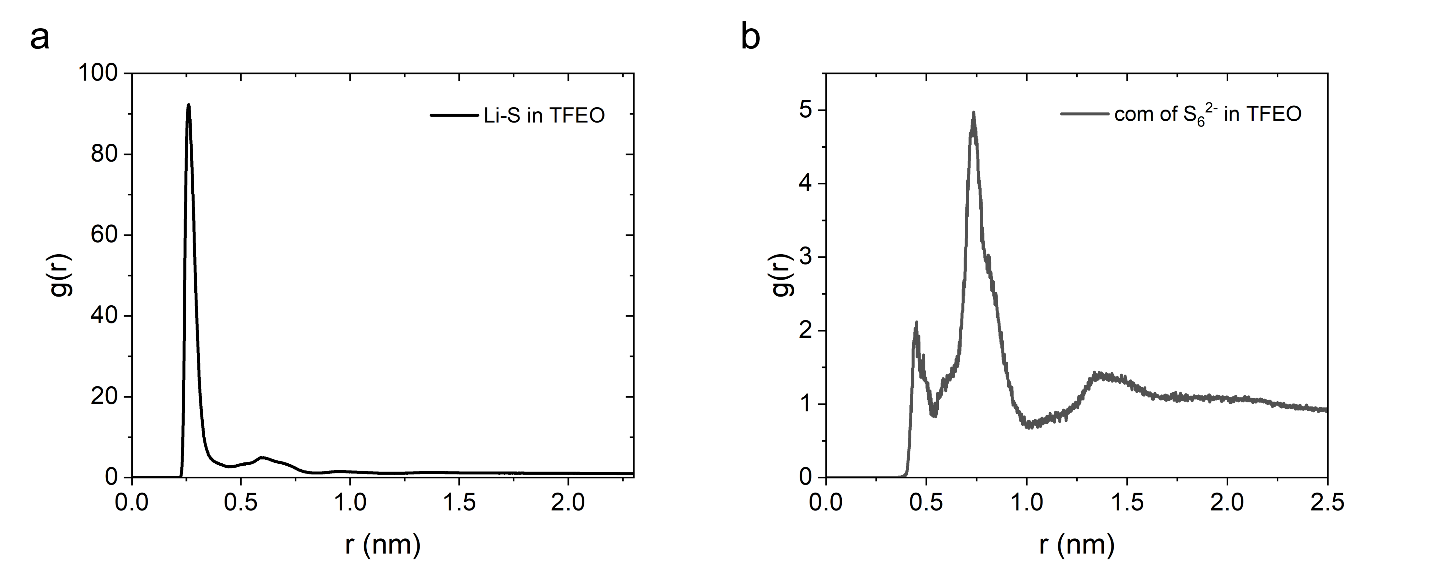


**Figure S13. (**a) RDF of Li-S bond in pure TFEO solvent. (b) RDF of center of mass (com) of S_6_^2-^ in pure TFEO solvent. Simulations contains 20 molecules of Li_2_S_6_ in the solution.

**Supplementary electrochemical performance of cycled cells**


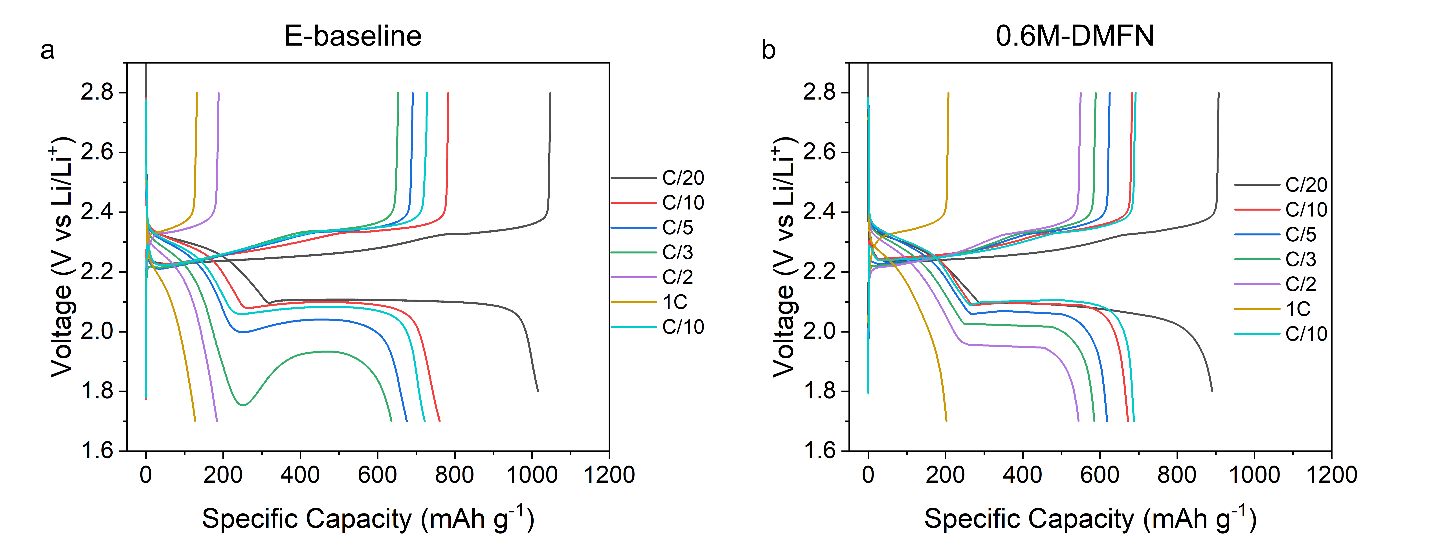


**Figure S14.** Voltage profiles of selected cycles for (a) E-baseline and (b) 0.6M-DMFN electrolytes of discharge rate capability testing which was cycled at varying discharge C rates.


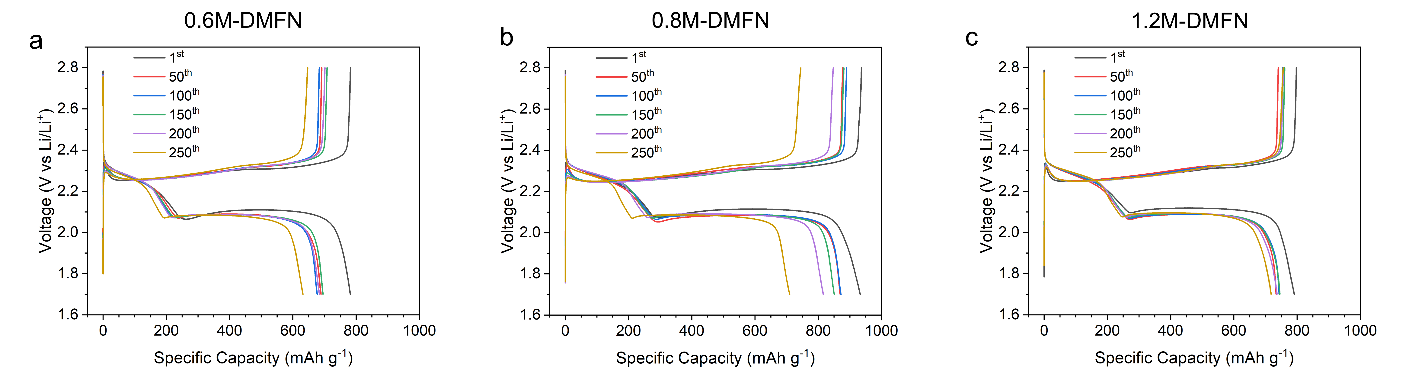


**Figure S15.** Voltage profiles of selected cycles for Li||S cells with (a) 0.6M-DMFN, (b) 0.8M-DMFN and (c) 1.2M-DMFN electrolytes cycled at C/5 rate. Li||S cells with an E/S ratio of 8 were first cycled at C/20 for 2 formation cycles (1.8 – 2.8 V) followed by C/5 rate (1.7 – 2.8 V) at 25 °C.


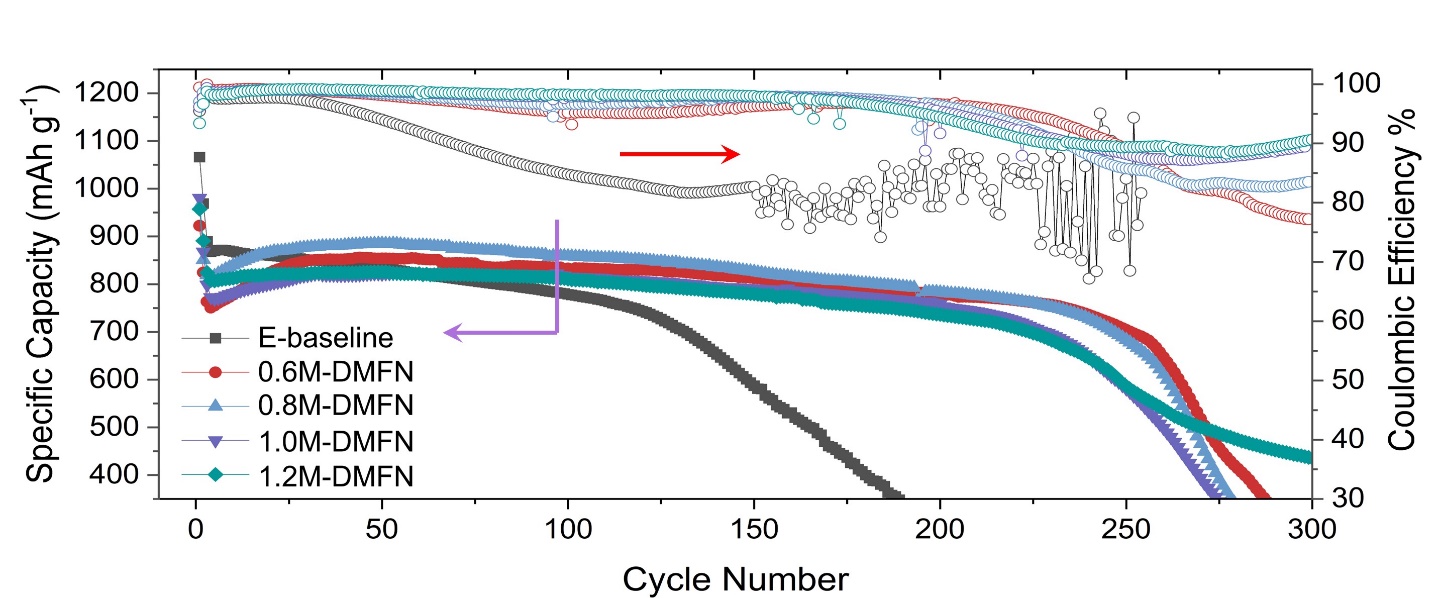


**Figure S16.** Cycling performance of LSBs using E-baseline and DMFN electrolytes at C/10 rate. All cells were cycled at 25 °C and LSBs were first cycled with 2 formation cycles at C/20 between 1.8 to 2.8 V followed by C/10 in the voltage range of 1.7-2.8 V.


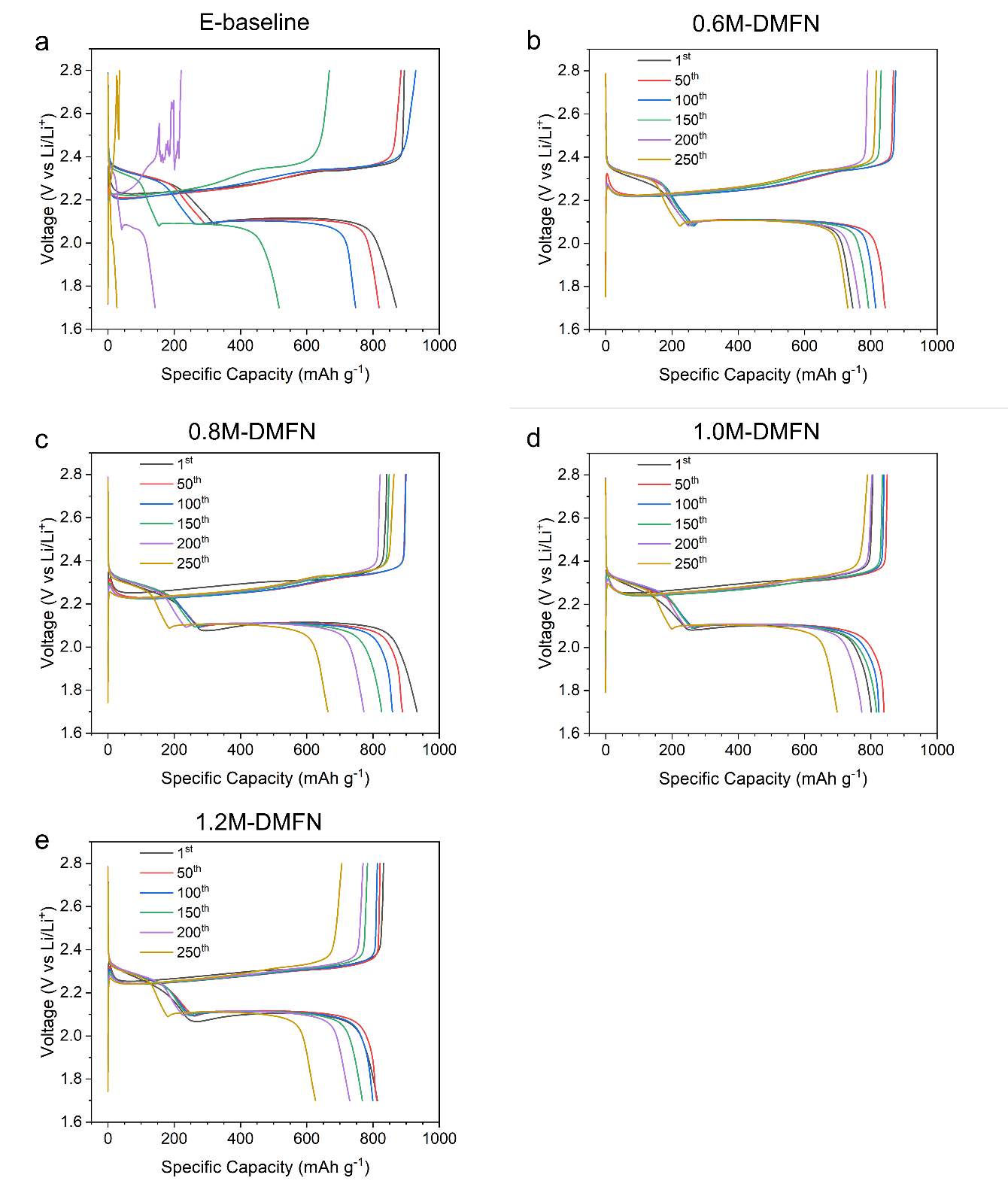


**Figure S17.** Voltage profiles of selected cycles for Li||S cells with (a) E-baseline, (b) 0.6M-DMFN, (c) 0.8M-DMFN, (d) 1.0M-DMFN and (e) 1.2M-DMFN electrolytes cycled at C/10 rate. Li||S cells with an E/S ratio of 8 were first cycled at C/20 for 2 formation cycles (1.8 – 2.8 V) followed by C/10 rate (1.7-2.8 V) at 25 °C.

**
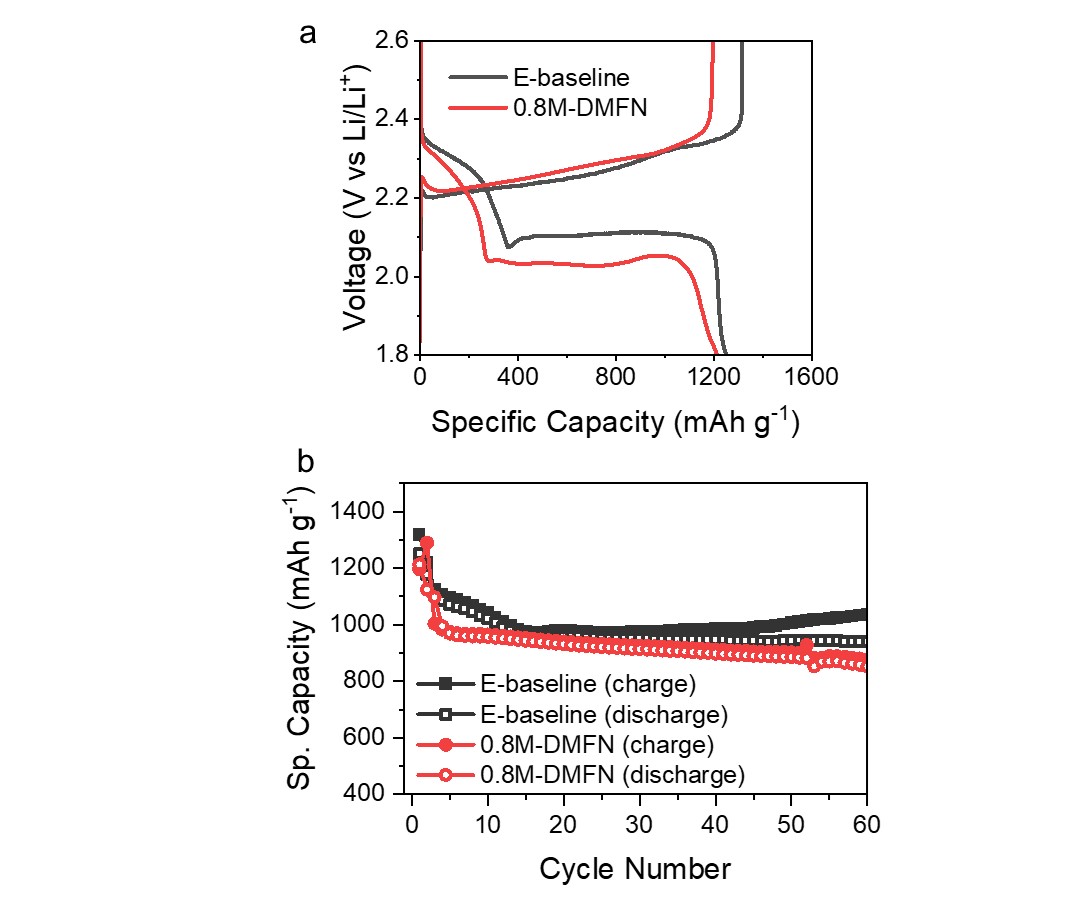
**

**Figure S18.** Performance of 1-Ah pouch cells of Li||S cells with E-baseline and 0.8M-DMFN electrolytes. (a) First cycle voltage profiles at C/20 rate. (b) Cycling performance at C/10 rate. The cells have S electrode loading of 4 mg-S cm^-2^, Li electrode of 100 µm thick, and E/S ratio at ~5.2 µL (mg-S)^-1^. The cells were first cycled at C/20 for 2 formation cycles in 1.8 – 2.6 V followed by regular cycling at C/10 rate in 1.7-2.6 V at 25 °C, where 1C = 1000 mA cm^-2^. The specific energy at C/20 of the two pouch cells without counting the pouch weight is 310 Wh kg^-1^ for E-baseline and 290 Wh kg^-1^ for 0.8M-DMFN. The lower specific energy of the 0.8M-DMFN cell is due to its lower initial specific capacity.

**Supplementary Characterization Results**


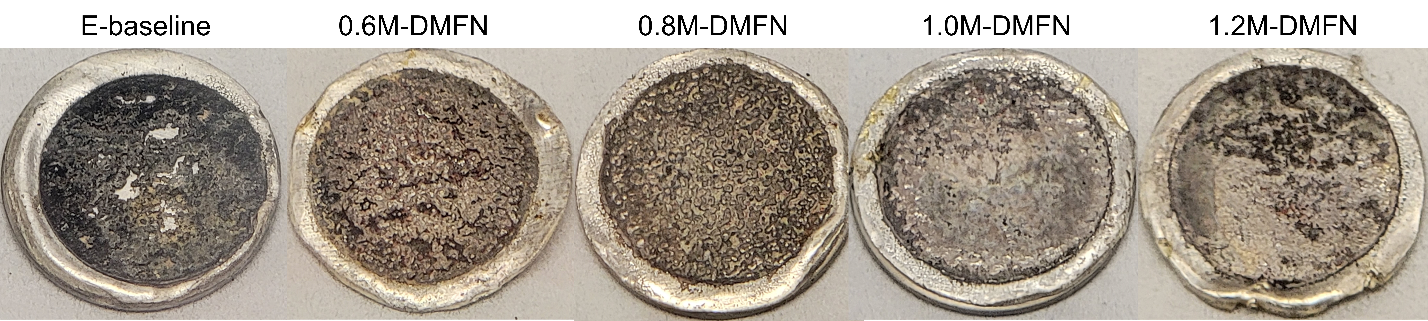


**Figure S19.** Photographs of LMAs cycled in different electrolytes after 300 cycles at C/5.


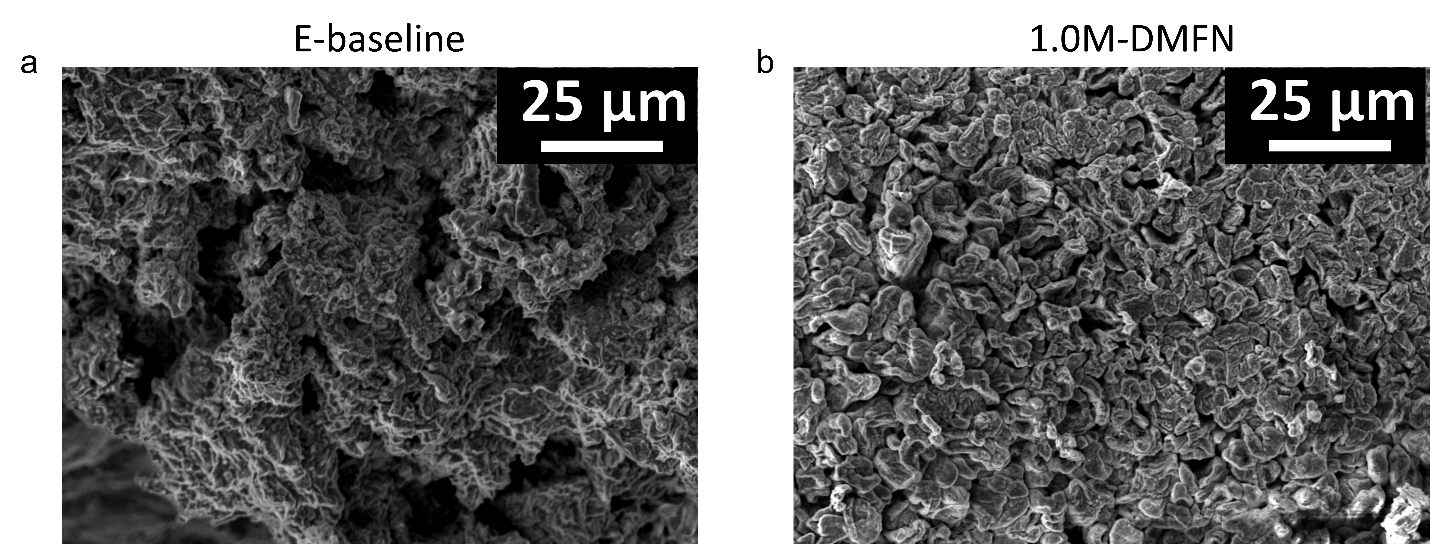


**Figure S20.** Top-down SEM images of cycled LMAs from Li||S cells with (a) E-baseline and (b) 1.0M-DMFN, respectively.


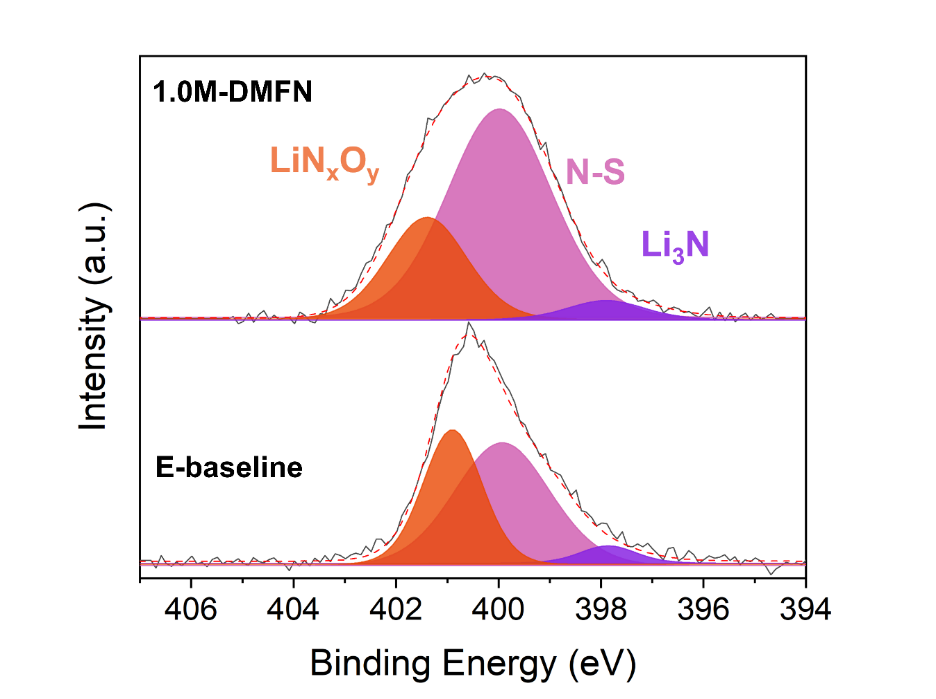


**Figure S21.** XPS analysis of N 1s spectra for LMAs from Li||S cells cycled in E-baseline and 1.0M-DMFN.


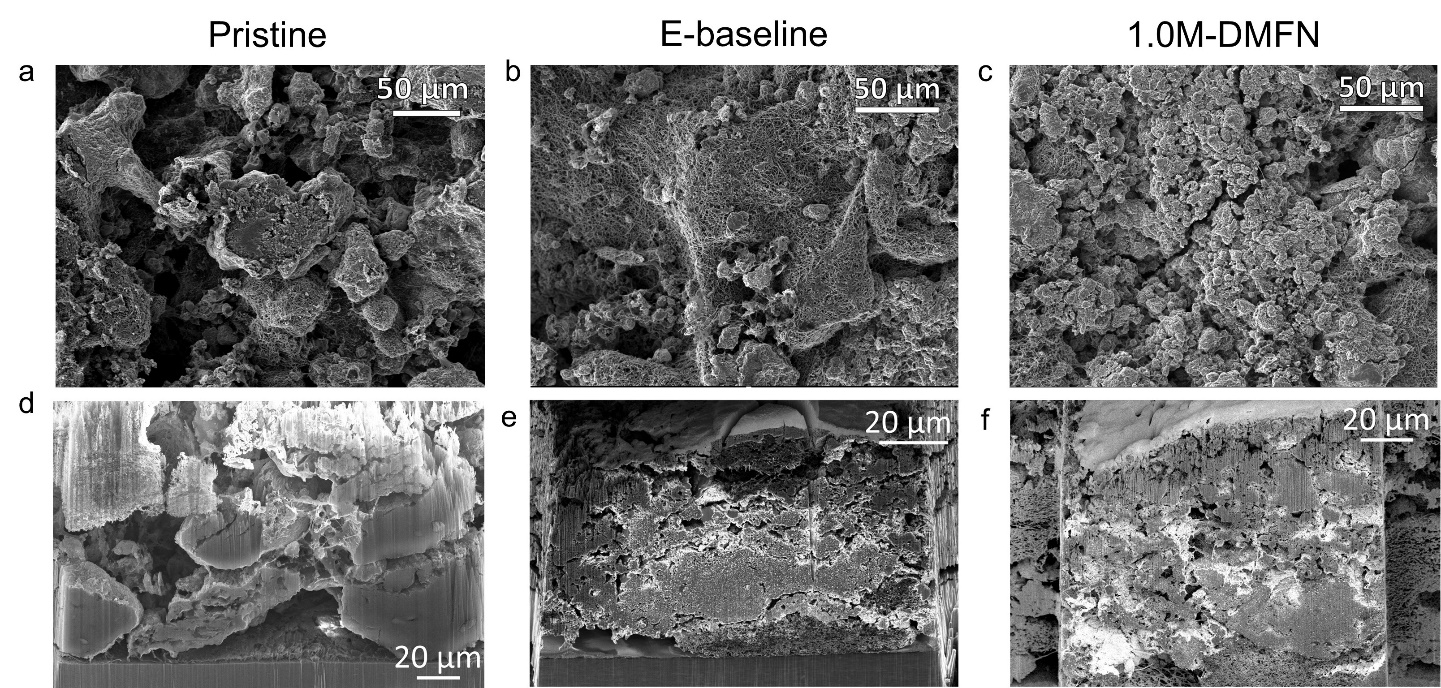
 **Figure S22.** SEM images of pristine S electrode and S electrodes after 300 cycles at C/5. (a-c) Top-down SEM images of pristine and cycled S electrodes from E-baseline and 1.0M-DMFN cells, respectively. (d-f) Cross-sectional plasma focused ion beam (PFIB)-SEM images of pristine and cycled S electrodes from E-baseline and 1.0M-DMFN cells, respectively.


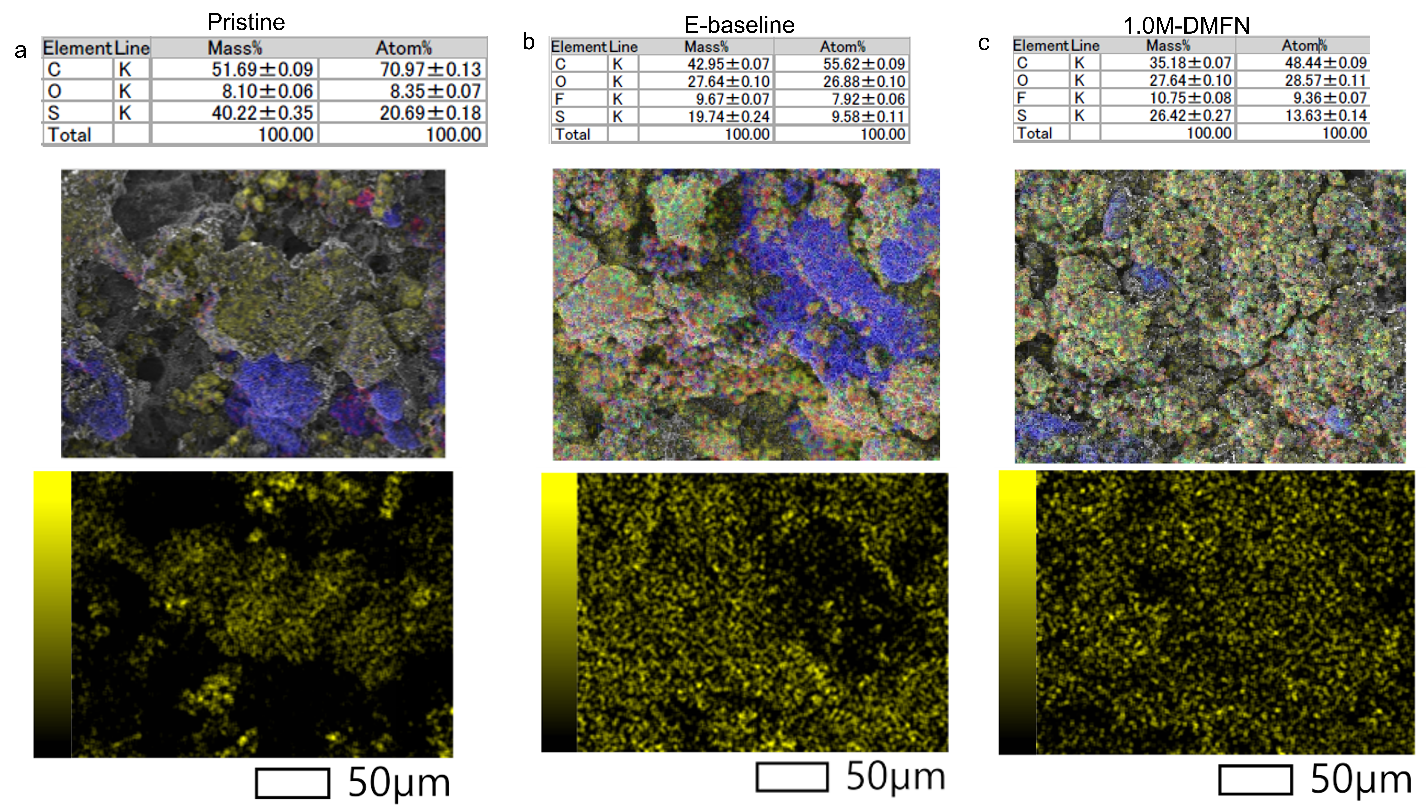


**Figure S23.** EDX analysis of top-down SEM images of (a) pristine S electrode and (b, c) cycled S electrodes from Li||S cells with E-baseline and 1.0M-DMFN, respectively. For color assignment, C = blue, O = red, F = green, and S = yellow.

**
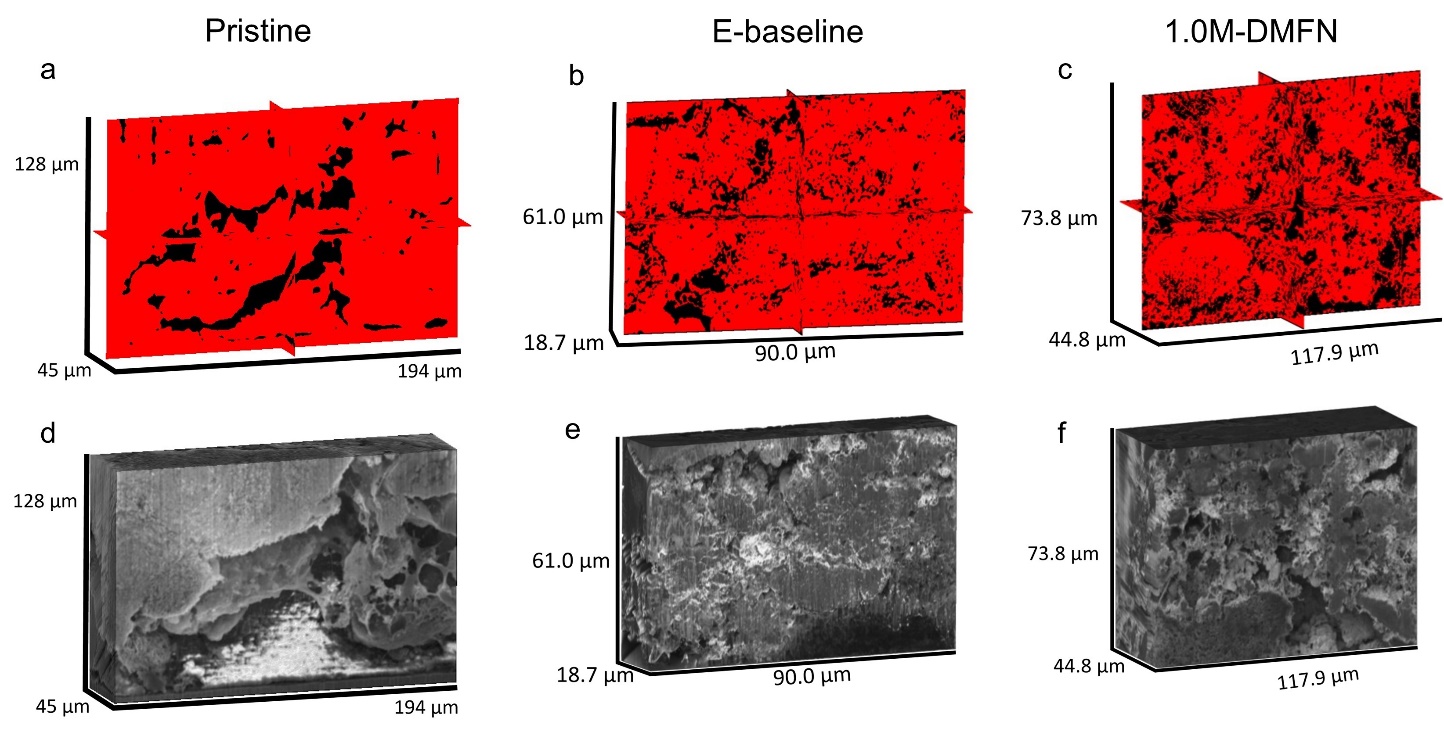
**

**Figure S24.** 3D reconstruction of PFIB-SEM data of pristine and cycled S electrodes. (a-c) Materials and voids and (d-f) all PFIB-SEM slices of (a, d) pristine S electrode, (b, e) cycled S electrode from Li||S cells with E-baseline, and (c, f) cycled S electrode from Li||S cells with 1.0M-DMFN.


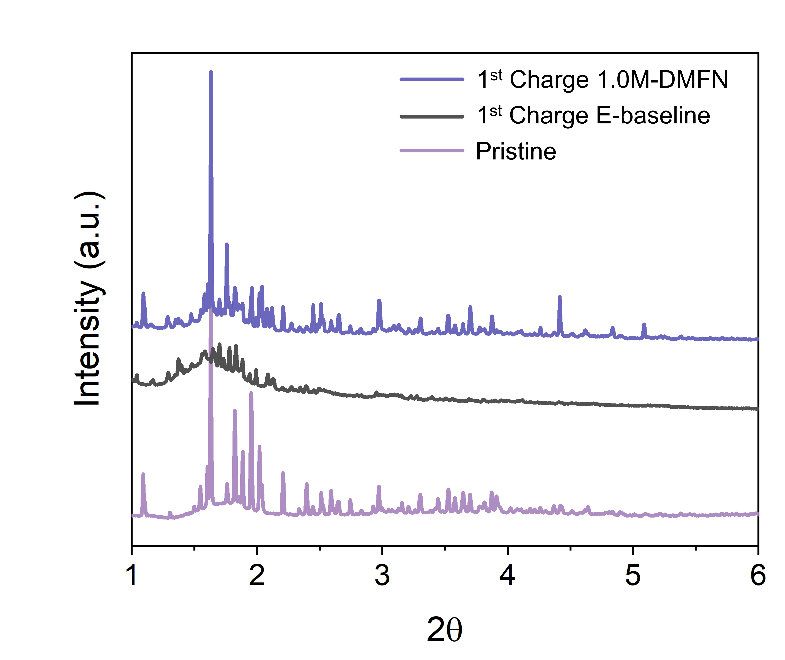


**Figure S25.** XRD analysis of S electrodes in pristine condition and after one complete formation cycle in the E-baseline and 1.0M-DMFN electrolytes.

**References**

[1] D. Lv, J. Zheng, Q. Li, X. Xie, S. Ferrara, Z. Nie, L. B. Mehdi, N. D. Browning, J. G. Zhang, G. L. Graff, J. Liu, J. Xiao, *Adv. Energy Mater.* **2015**, *5*, 1402290.

[2] B. D. Adams, J. Zheng, X. Ren, W. Xu, J. G. Zhang, *Adv. Energy Mater.* **2018**, *8*, 1702097.

[3] G. Bussi, D. Donadio, M. Parrinello, *J. Chem. Phys.* **2007**, *126*, 014101.

[4] H. J. C. Berendsen, J. P. M. Postma, W. F. Van Gunsteren, A. Dinola, J. R. Haak, *J. Chem. Phys.* **1984**, *81*, 3684.

[5] S. Nosé, *J. Chem. Phys.* **1984**, *81*, 511.

[6] T. Darden, D. York, L. Pedersen, *J. Chem. Phys.* **1993**, *98*, 10089.

[7] B. Hess, H. Bekker, H. J. C. Berendsen, J. G. E. M. Fraaije, *J. Comput. Chem.* **1997**, *18*, 1463.

[8] W. Humphrey, A. Dalke, K. Schulten, *J. Mol. Graphics* **1996**, *14*, 33.

[9] J.-M. Kim, P. Gao, Q. Miao, Q. Zhao, M. M. Rahman, P. Chen, X. Zhang, E. Hu, P. Liu, J.-G. Zhang, W. Xu, *ACS Appl. Mater. Interfaces* **2024**, *16*, 20618.

[10] T. Tran, X. Cao, Y. Xu, P. Gao, H. Zhou, F. Guo, K. S. Han, D. Liu, P. M. Le, J. M. Weller, M. H. Engelhard, C. Wang, M. S. Whittingham, W. Xu, J.-G. Zhang, *Adv. Funct. Mater.* **2024**, *34*, 2407012.

[11] C. Prescher, V. B. Prakapenka, *High Pressure Research* **2015**, *35*, 223.

[12] P. Juhás, T. Davis, C. L. Farrow, S. J. L. Billinge, *J. Appl. Crystal.* **2013**, *46*, 560.

[13] L. X. Dang, *J. Chem. Phys.* **1992**, *96*, 6970.

[14] J. N. Canongia Lopes, A. A. H. Pádua, *J. Phys. Chem. B* **2004**, *108*, 16893.

[15] N. N. Rajput, V. Murugesan, Y. Shin, K. S. Han, K. C. Lau, J. Chen, J. Liu, L. A. Curtiss, K. T. Mueller, K. A. Persson, *Chem. Mater.* **2017**, *29*, 3375.

[16] A. Andersen, N. N. Rajput, K. S. Han, H. Pan, N. Govind, K. A. Persson, K. T. Mueller, V. Murugesan, *Chem. Mater.* **2019**, *31*, 2308.

[17] L. S. Dodda, J. Z. Vilseck, J. Tirado-Rives, W. L. Jorgensen, *J. Phys. Chem. B* **2017**, *121*, 3864.

[18] L. S. Dodda, I. Cabeza de Vaca, J. Tirado-Rives, W. L. Jorgensen, *Nucleic Acids Research* **2017**, *45*, W331.

[19] C. Park, M. Kanduč, R. Chudoba, A. Ronneburg, S. Risse, M. Ballauff, J. Dzubiella, *J. Power Sources* **2018**, *373*, 70.

[20] C. Park, A. Ronneburg, S. Risse, M. Ballauff, M. Kanduč, J. Dzubiella, *J. Phys. Chem. C* **2019**, *123*, 10167.

[21] G. M. C. Silva, P. Morgado, P. Lourenço, M. Goldmann, E. J. M. Filipe, *Proc. Natl. Acad. Sci. U.S.A.* **2019**, *116*, 14868.

[22] C. Park, M. Kanduč, T. F. Headen, T. G. A. Youngs, J. Dzubiella, S. Risse, *Energy Storage Mater.* **2022**, *52*, 85.

[23] I. V. Leontyev, A. A. Stuchebrukhov, *J. Chem. Phys.* **2009**, *130*, 085102.

[24] J. C. R. Reis, I. M. S. Lampreia, Â. F. S. Santos, M. L. C. J. Moita, G. Douhéret, *ChemPhysChem* **2010**, *11*, 3722.

[25] R. Francesconi, A. Bigi, K. Rubini, F. Comelli, *J. Chem. Eng. Data* **2007**, *52*, 2020.

[26] F. Comelli, S. Ottani, R. Francesconi, C. Castellari, *J. Chem. Eng. Data* **2002**, *47*, 1226.

[27] L. Sarkar, M. N. Roy, *J. Chem. Eng. Data* **2009**, *54*, 3307.

[28] M. Makosza, B. Jerzak, M. Fedorynski, *Roczniki Chemii* **1975**, *49*, 1783.
